# Phenotypic and genomic profiling of multidrug-resistant *Escherichia coli *and* Klebsiella pneumoniae* isolated from Intensive Care Unit patients in Kenya

**DOI:** 10.1186/s12866-026-04880-5

**Published:** 2026-03-23

**Authors:** Beatrice Oduor, Anne Maina, Job Mwale, Brian Ogoti, Leon Otieno, Moureen Jepleting, Charchil Ayodo, Robert Mugoh, Teresa Ita, Josiah Kuja, Sylvia Omulo

**Affiliations:** 1 Department of Medical Microbiology and Immunology, University of Nairobi, Nairobi, Kenya; 2 Washington State University Global Health–Kenya, Nairobi, Kenya; 3 Kenyatta National Hospital, Nairobi, Kenya; 4 Centre for Epidemiological Modelling and Analysis, University of Nairobi, Nairobi, Kenya; 5 Paul G. Allen School for Global Health, Washington State University, Pullman, WA USA; 6 University of Nairobi Institute of Tropical and Infectious Diseases, Nairobi, Kenya

**Keywords:** Antimicrobial resistance, Carbapenemase, ESBL, Intensive care unit, Resistance genes, Virulence factors, Whole-genome sequencing

## Abstract

**Background:**

The global rise of multidrug-resistant Enterobacterales, particularly extended-spectrum beta-lactamase (ESBL)- and carbapenemase-producing *Escherichia coli* and *Klebsiella pneumoniae*, poses a critical threat to intensive care unit (ICU) patients. Despite the clinical importance of these pathogens in sub-Saharan Africa, studies on their molecular characterization remains scarce.

**Methods:**

We performed antibiotic susceptibility testing and whole-genome sequencing (WGS) on 22 multidrug-resistant isolates (*15 E. coli* and 7 *K. pneumoniae*) recovered from ICU patients at Kenya’s public referral hospital between January and June 2021. Isolates were obtained from blood, tracheal aspirates, urine, and pus swabs. We characterized antimicrobial resistance genes, virulence determinants, plasmid replicons, and genetic lineages, and assessed phenotype-genotype concordance.

**Results:**

All isolates exhibited resistance to third-generation cephalosporins, with resistance rates exceeding 80% for at least 10 antibiotics. The ESBL gene *bla*_CTX−M−15_ was detected in 93% of *E. coli* and 100% of *K. pneumoniae* isolates. The concordance between phenotypic resistance and genotypic determinants was highest for β-lactams and lowest for aminoglycosides, trimethoprim-sulfamethoxazole and carbapenems. MLST revealed considerable genetic diversity among *E. coli*, including high-risk clones STc131 and ST648. Virulence genes (e.g., *fimH*, *chuA*, *fyuA*) were more prevalent in *E. coli*, particularly among isolates from urine and tracheal aspirate samples. Plasmid profiling revealed greater replicon diversity in *E. coli*, with frequent detection of IncFII and IncFIB plasmids, suggesting a stronger capacity for horizontal gene transfer.

**Conclusion:**

These findings underscore the complexity of multidrug-resistant pathogen dynamics in ICU environments. They emphasize the need for species-specific surveillance, rapid diagnostics, and context-informed stewardship strategies to guide infection control and therapeutic interventions. While limited in scale, this study adds to the genomic landscape of multidrug-resistant pathogens in African ICUs and highlights critical targets for infection control and therapeutic interventions.

**Supplementary Information:**

The online version contains supplementary material available at 10.1186/s12866-026-04880-5.

## Introduction 

The global rise of antimicrobial resistance (AMR) poses a serious threat to public health, undermining efforts to treat infectious diseases in both humans and animals [1]. Of particular concern is the growing prevalence of extended-spectrum beta-lactamase (ESBL)- and carbapenemase-producing *Enterobacterales*, such as *E. coli*, and *K. pneumoniae*, which are associated with increased morbidity, prolonged hospital stays, and higher treatment costs [1]. In 2019 alone, AMR was implicated in 1.27 million deaths globally, exceeding combined fatalities from malaria and HIV [2]. The burden is disproportionately higher in low- and middle-income countries, with sub-Saharan Africa experiencing AMR-related deaths at nearly four times the rate observed in high-income regions [2]. Without substantial mitigation strategies, drug-resistant infections are projected to claim up to 10 million lives annually by 2050 [3].

The surge of multidrug-resistant organisms represents a critical challenge for healthcare systems, particularly in intensive care units (ICUs), where patients face increased risk of severe infections [4]. Against this global backdrop, Kenya faces particularly acute challenges with widespread circulation of multidrug-resistant Gram-negative bacteria—including *E. coli* and *K. pneumoniae*—in both hospital and community settings [4–6]. A 2022 study highlighted a high prevalence of ESBL-producing *Enterobacterales* colonization among hospitalized patients, as well as the emergence of carbapenemase-producing strains [7]. However, most surveillance efforts remain limited to phenotypic susceptibility testing, offering little insight into the molecular characteristics of circulating strains.

*Enterobacterales* are particularly problematic due to their capacity to rapidly acquire and horizontally transfer resistance genes, complicating treatment and contributing to high mortality [8, 9]. Among these pathogens, high-risk clones are of particular concern as they often possess traits such as toxin production, adhesion, and immune evasion, leading to more severe infections and limited therapeutic options [10]. Despite their clinical relevance, data on the molecular epidemiology, genetic diversity, and infection site-specific resistance and virulence profiles of multidrug-resistant *Enterobacterales* in Kenyan ICUs remain scarce.

We analyzed multidrug-resistant ESBL- and/or carbapenemase-producing *E. coli* and *K. pneumoniae* isolates recovered from ICU patients at Kenya’s largest public referral hospital. Using WGS, we characterized their AMR genes, virulence determinants, plasmid replicons, and genetic lineages. By integrating clinical metadata to assess resistance and virulence burdens across infection sites, this study explored potential niche-specific adaptations, generating insights that can strengthen AMR surveillance and guide infection control strategies in resource-limited critical care settings.

## Materials and methods

### Sample collection and bacterial isolation

Multidrug-resistant ESBL- and/or carbapenemase-producing *E. coli* and *K. pneumoniae* isolates were selected from a larger surveillance study involving ICU patients at the Kenyatta National Hospital (KNH) who had clinically suspected bacterial infections between January and June 2021 [11]. The study targeted patients admitted to any ICU in KNH with a suspected bacterial infection during their hospitalization. Patients with at least one gram-negative culture-positive sample were eligible for participation. Samples included blood, tracheal aspirates, urine, and pus swabs. Blood samples were cultured in blood culture bottles and incubated at 35–37 °C overnight. Subsequent sub-culturing was performed on blood agar, chocolate agar, and MacConkey agar. Tracheal aspirates and pus swabs were also cultured on the same media, while urine samples were inoculated on CLED agar. Blood and chocolate agar plates were incubated in 5–10% CO₂ at 35–37 °C; MacConkey and CLED plates were incubated aerobically under the same conditions.

### Bacterial identification and antimicrobial susceptibility testing

Isolates were identified using the Vitek®2 automated system (Biomérieux, France) with GN83 Gram-negative identification cards. A panel of 18 antibiotics was tested including: ampicillin, amoxicillin/clavulanate, amikacin, aztreonam, ceftazidime, cefazolin, ciprofloxacin, ceftriaxone, cefotaxime, cefuroxime, cefuroxime/axetil, cefepime, gentamicin, meropenem, ampicillin/sulbactam, sulfamethoxazole/ trimethoprim, piperacillin/tazobactam, Nitrofurantoin.

Antimicrobial susceptibility testing (AST) was performed according to Clinical and Laboratory Standards Institute 2020 guidelines [12]. Bacterial suspensions were prepared in 0.5% saline and adjusted to a 0.5 McFarland turbidity standard before testing. All isolates identified as ESBL-producing and carbapenemase-producing were archived in skimmed milk-tryptone-glucose-glycerol broth at − 80 °C for subsequent molecular characterization [11].

### DNA extraction and whole-genome sequencing (WGS)

Stored multidrug-resistant isolates were revived on MacConkey agar and incubated overnight at 37 °C. Single colonies were inoculated into Tryptic Soy Broth and incubated at 37 °C for 18–24 h. Genomic DNA was extracted using the Qiagen DNeasy UltraClean Microbial Kit (Qiagen, Germany) per the manufacturer’s instructions [13]. DNA concentration was quantified using the Qubit™ 4 fluorometer (Thermo Fisher Scientific, USA) and normalized to a minimum concentration of 50 ng/µL for sequencing. ATCC BAA-196 (*E. coli*) and ATCC 700,603 (*K. pneumoniae*) were used as positive controls, and DNA/RNA-free water served as a negative control during DNA extraction and library preparation.

### Library preparation and nanopore sequencing

A DNA library was prepared using the Oxford Nanopore Technologies (ONT) MinION platform. DNA underwent end-repair, barcoding, and adapter ligation using the SQK-LSK109 genomic sequencing kit and the EXP-NBD104/114 native barcoding expansion kits (ONT, UK). Purification was performed using AMPure XP magnetic beads (Beckman Coulter, USA). The pooled library was then loaded onto a MinION flow cell and sequenced according to the manufacturer’s protocol [14].

### Bioinformatics and genomic analyses

Basecalling was performed using the Guppy basecaller Guppy version 6.4.6, adapter trimming together with demultiplexing was carried out using Porechop version 0.2.4 [15]. *De novo* genome assembly was carried out using Canu version 2.2 [16], and assembly quality was assessed using Quast version 5.2.0 [17], the assembly metrics are provided (Supplementary Table S3). We used AMRFinderPlus version 4.0.23 with database version 2025-07-16.1 to identify antimicrobial resistance genes with ≥ 90% identity and ≥ 90% coverage [18], multilocus sequence typing (MLST), virulence gene detection were carried out using the Bacterial Isolate Genome Sequence Database (BIGSdb-Pasteur) version 1.41.0 [19] and plasmids identified using plasmid finder version 2.1 [20].

### Data analysis

Multidrug resistance status was determined based on resistance to at least one agent in three or more antimicrobial classes. ESBL-producing isolates were defined as those susceptible to meropenem but resistant to ceftriaxone. Isolates resistant to meropenem were considered carbapenemase-producers while resistant to all antibiotics tested were classified as pan-resistant. For each isolate–antibiotic combination, phenotypic resistance was matched against the presence of known resistance genes associated with that drug class. Concordance was classified as: Concordant (+/+) if both resistance and gene were present; Concordant (–/–) if neither was present; Pheno+/Geno– if phenotypically resistant but lacking a known gene; or Pheno–/Geno + if phenotypically susceptible despite harboring resistance genes. This was done using epiR package in R (version 4.2.2).

## Results

### Antibiotic susceptibility

**Fig. 1:**
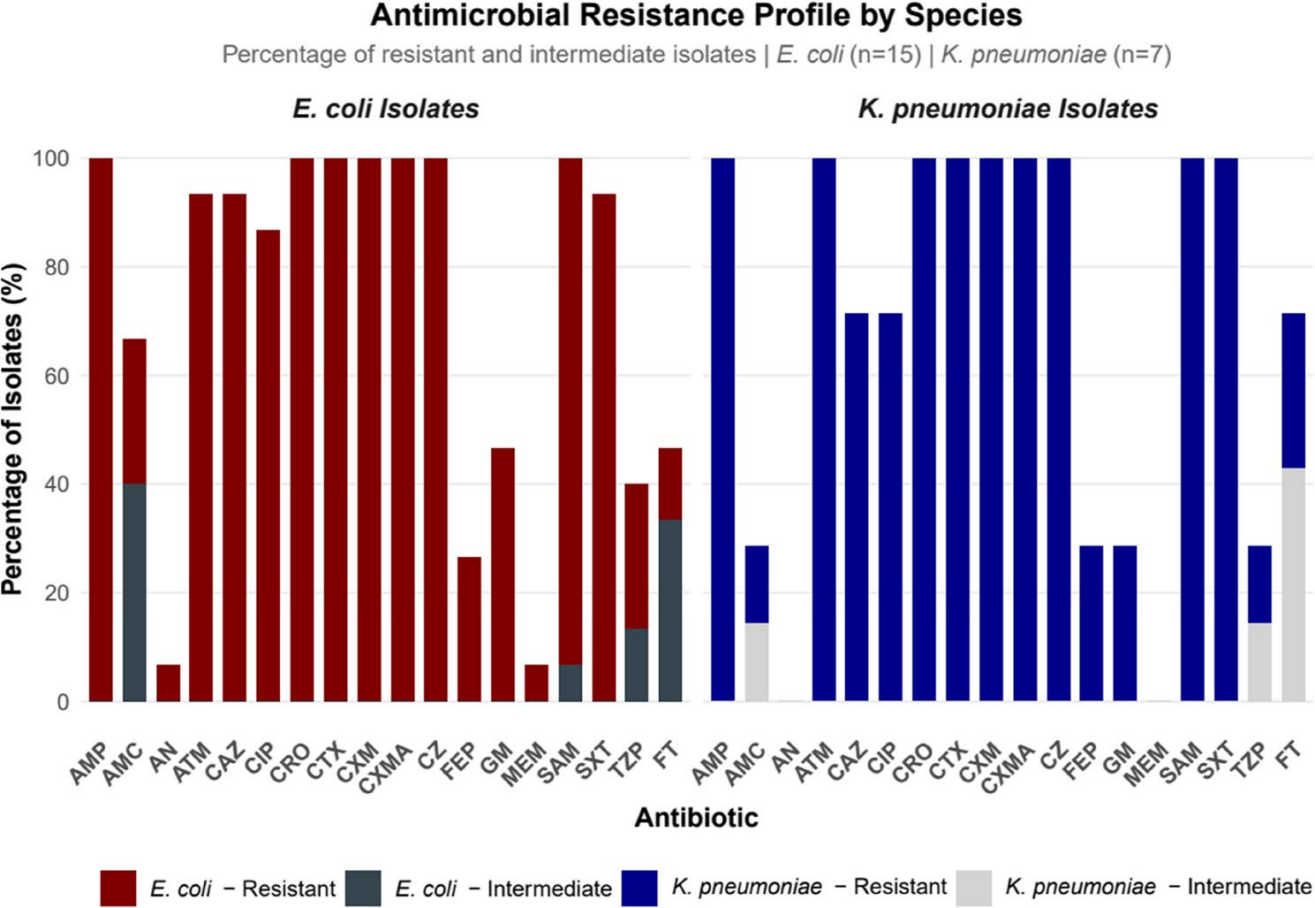
Antimicrobial resistance profiles 18 antibiotics among multidrug-resistant *E. coli* (*n* = 15) and *K. pneumoniae* (*n* = 7) isolates. AMP: ampicillin; AMC: amoxicillin/clavulanate; AMK: amikacin; ATM: aztreonam; CAZ: ceftazidime; CEZ: cefazolin; CIP: ciprofloxacin; CRO: ceftriaxone; CTX: cefotaxime; CXM: cefuroxime; CXMA: cefuroxime/axetil; FEP: cefepime; GEN: gentamicin; MEM: meropenem; SAM: ampicillin/sulbactam; SXT: sulfamethoxazole/ trimethoprim; TZP: piperacillin/tazobactam; FT: Nitrofurantoin

A total of 22 multidrug-resistant isolates, 15 *E. coli* and 7 *K. pneumoniae* were analyzed. *E. coli* isolates were obtained from tracheal aspirates (*n* = 8), urine (*n* = 5), pus swab (*n* = 1), and blood (*n* = 1), while *K. pneumoniae* isolates were recovered from tracheal aspirates (*n* = 3), urine (*n* = 2), pus swab (*n* = 1), and blood (*n* = 1). All isolates were resistant to ampicillin, cefuroxime, ceftriaxone, cefotaxime, and cefazolin (Fig. 1). All *K. pneumoniae* isolates were susceptible to amikacin and meropenem. Resistance rates exceeded 80% for at least 10 antibiotics across the isolates. Lower resistance frequencies (< 30%) were observed for amoxicillin/clavulanate (AMC), cefepime (FEP), gentamicin (GEN), and piperacillin/tazobactam (TZP), especially among *K. pneumoniae* isolates (Fig. 1).

### Multidrug-resistance patterns

The 22 isolates exhibited 10 distinct resistance profiles, with individual isolates resistant to 9–18 antibiotics. The profiles varied by species (Table 1). Five of these profiles were exclusive to *E. coli*, two were unique to *K. pneumoniae*, while the remaining were shared. Among *E. coli* isolates, eight (53%) were resistant to 11 antibiotics, three (20%) to 12 antibiotics, two (13%) to 16 antibiotics, and one isolate to each of the 18 antibiotics. Among *K. pneumoniae*, three (43%) isolates were resistant to 11 antibiotics, one (14%) to 12 antibiotics, two (29%) to 10 antibiotics, and one to 16 antibiotics. The most common profile in *E. coli* consisting of resistance to ampicillin, aztreonam, ceftazidime, cefazolin, ciprofloxacin, ceftriaxone, cefotaxime, cefuroxime, cefuroxime/axetil, ampicillin/sulbactam, and sulfamethoxazole/trimethoprim was found in five isolates and was also one of the two most common profiles observed in *K. pneumoniae* (Table 1).

Tracheal aspirates had the greatest diversity of multidrug resistance profiles, with eight distinct patterns identified (Table 1). Urine samples accounted for six profiles, while blood and pus swabs had two and one profiles, respectively. Three profiles were observed across multiple sample types. One profile was shared by isolates from urine, tracheal aspirate, and pus swab; a second profile was detected in both blood and tracheal aspirate isolates; and a third profile was found in urine and tracheal aspirates. Other patterns were restricted to single sample types. The only isolate that exhibited resistance to all 18 antibiotics tested was recovered from a urine sample (Table 1). 

**Table 1:** Multidrug resistance profiles identified among *E. coli* (*n* = 15) and *K. pneumoniae* (*n* = 7) isolates

Multidrug-resistance profile	E. coli	K. pneumo	#ABx
AMP-AMC-AMK-ATM-CAZ-CEZ-CIP-CRO-CTX-CXM-CXMA-FEP-GEN-MEM-SAM-SXT-TZP-FT	1 (U)	0	18
AMP-AMC-ATM-CAZ-CEZ-CIP-CRO-CTX-CXM-CXMA-FEP-GEN-SAM-SXT-TZP-FT	2 (T)	1 (U)	16
AMP-AMC-ATM-CAZ-CEZ-CRO-CTX-CXM-CXMA-SAM-TZP	1 (B)	0	11
AMP-ATM-CAZ-CEZ-CIP-CRO-CTX-CXM-CXMA-FEP-SXT	1 (T)	0	11
AMP-ATM-CAZ-CEZ-CIP-CRO-CTX-CXM-CXMA-GEN-SAM-SXT	3 (2T, 1U)	0	12
AMP-ATM-CAZ-CEZ-CIP-CRO-CTX-CXM-CXMA-SAM-SXT	5 (1P, 1T, 3U)	2 (1P, 1T)	11
AMP-ATM-CAZ-CEZ-CRO-CTX-CXM-CXMA-FEP-SAM-SXT-FT	0	1 (T)	12
AMP-ATM-CAZ-CEZ-CRO-CTX-CXM-CXMA-GEN-SAM-SXT	1 (T)	1 (B)	11
AMP-ATM-CEZ-CIP-CRO-CTX-CXM-CXMA-SAM-SXT	0	2 (T, U)	10
AMP-CEZ-CIP-CRO-CTX-CXM-CXMA-SAM-SXT	1 (T)	0	9

Each row represents a unique resistance profile defined by the combination of antibiotics to which resistance was observed. Isolate counts for each species are shown along with their corresponding sample types: B = blood, P = pus swab, T = tracheal aspirate, U = urine. The total number of antibiotics in each profile is indicated under “#Abx”. Antibiotics: AMP, ampicillin; AMC, amoxicillin/clavulanate; AMK, amikacin; ATM, aztreonam; CAZ, ceftazidime; CEZ, cefazolin; CIP, ciprofloxacin; CRO, ceftriaxone; CTX, cefotaxime; CXM, cefuroxime; CXMA, cefuroxime/axetil; FEP, cefepime; GEN, gentamicin; MEM, meropenem; SAM, ampicillin/sulbactam; SXT, sulfamethoxazole/trimethoprim; TZP, piperacillin/tazobactam; FT: nitrofurantoin

### Resistance gene profiles

**Fig. 2:**
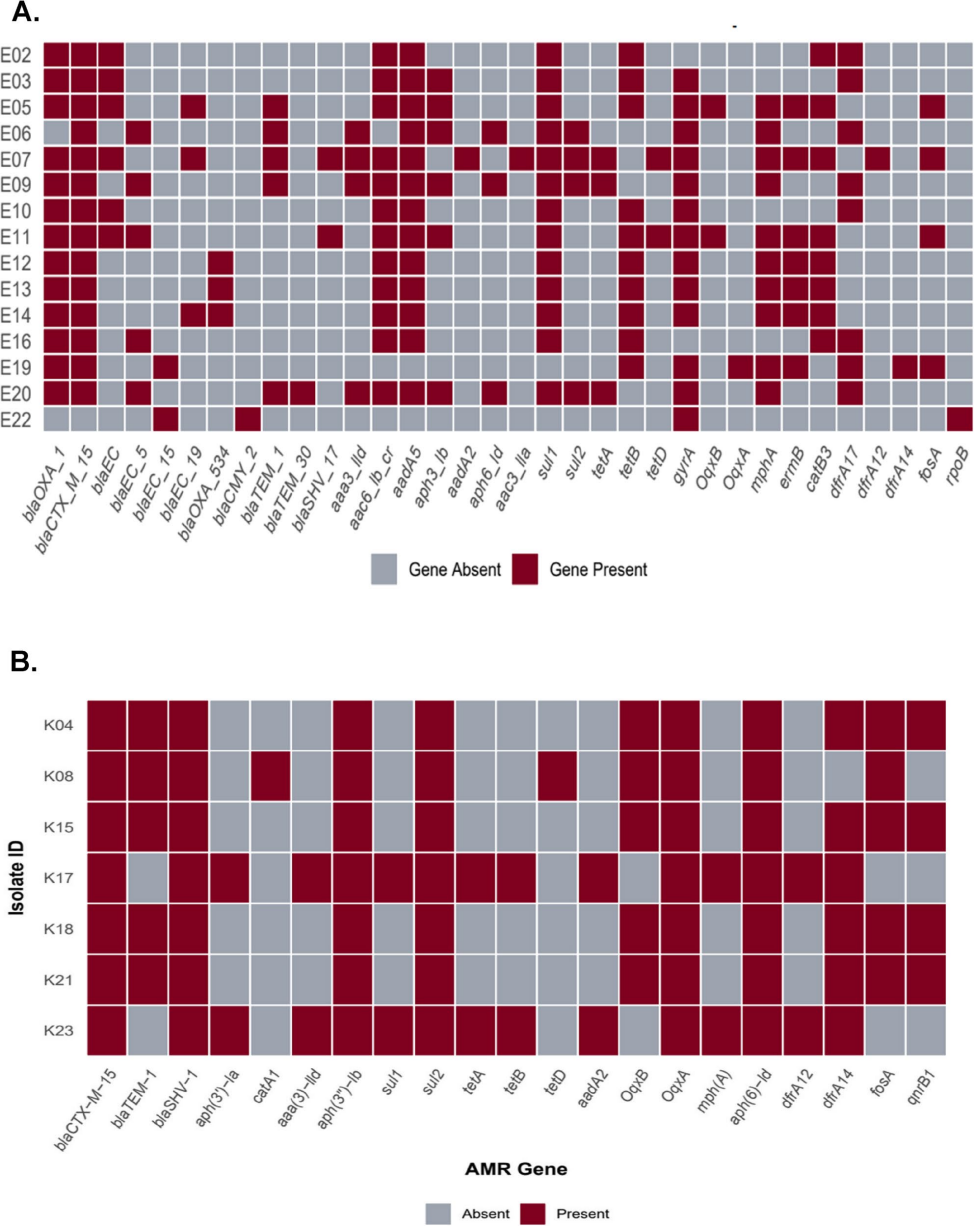
**A**. Heatmap showing the AMR genes detected among the *E. coli* (*n* = 15) isolates. **B** Heatmap showing the AMR genes detected among the K. pneumoniae (n=7) isolates

*E. coli* isolates harbored 28 distinct AMR genes (Fig. 2A), with β-lactamase genes being most prevalent. Notably, *bla*_CTX−M−15_ and *bla*_OXA−1_ were detected in 93% and 87% of isolates, respectively. Other frequently detected genes included aminoglycoside resistance genes (*aadA5*: 87%, *aac(6’)-Ib-cr*: 80%), sulfonamide resistance genes (*sul1*: 87%), and tetracycline resistance genes (*tetB*: 67%). Complete gene frequencies are shown in Fig. 2A and B.

The heatmap illustrates the distribution of 21 AMR genes across seven *K. pneumoniae* isolates. All isolates (7/7, 100%) carried the ESBL gene *bla*_*CTX−M−15*_. Other prevalent genes, detected in 71–86% of isolates, included *bla*_*TEM−1*_ (5/7), *aph* [6]*-Id* (7/7), *sul2* (7/7), *dfrA14* (6/7) and *qnrB1* (4/7). Genes detected in only one or two isolates (14–29%) included *tetA/B/D*, *catA1*, *aac(3’)-IId*, *aph(3’)-Ia*, *sul1*, *dfrA12*, as well as *aadA2* (2/7) and *mphA* (2/7).

### Phenotype–genotype concordance

**Fig. 3:**
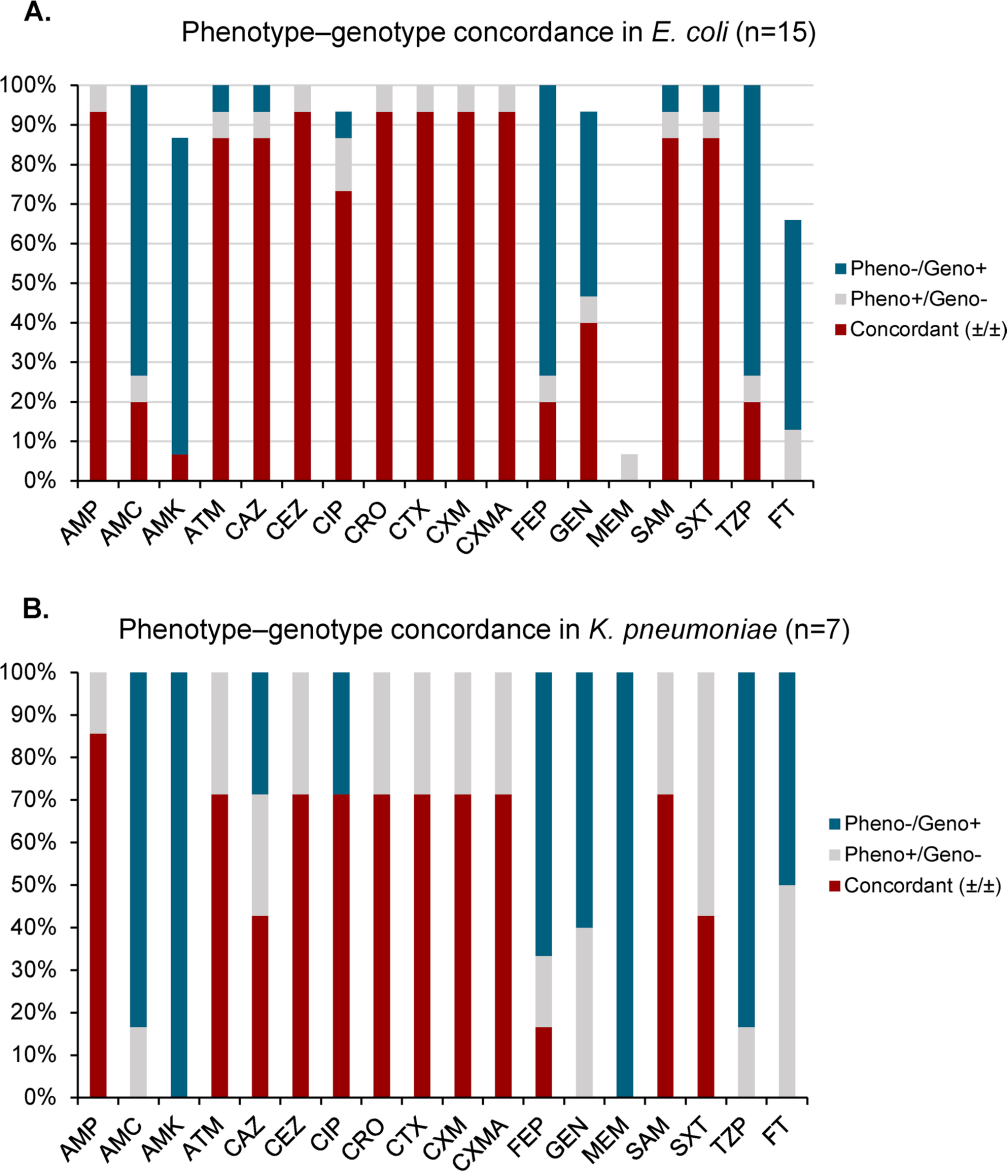
**A**. Phenotype–genotype concordance in *E. coli* (*n* = 15). **B **Phenotype–genotype concordance in K. pneumoniae (n=7). Bar plots show the proportion of isolates for each antibiotic categorized as: concordant (both phenotypically resistant and carrying ≥ 1 corresponding resistance gene, or phenotypically susceptible and lacking those genes); phenotypically resistant without the corresponding gene(s) (Pheno+/Geno–); or carrying resistance gene(s) without phenotypic resistance (Pheno–/Geno+). Anibiotics: AMP: ampicillin; AMC: amoxicillin/clavulanate; AMK: amikacin; ATM: aztreonam; CAZ: ceftazidime; CEZ: cefazolin; CIP: ciprofloxacin; CRO: ceftriaxone; CTX: cefotaxime; CXM: cefuroxime; CXMA: cefuroxime/axetil; FEP: cefepime; GEN: gentamicin; MEM: meropenem; SAM: ampicillin/sulbactam; SXT: sulfamethoxazole/ trimethoprim; TZP: piperacillin/tazobactam; FT: nitrofurantoin

To assess the alignment between phenotypic resistance and underlying genotypic determinants, we evaluated phenotype–genotype concordance for all 22 isolates across 18 antibiotics (Fig. 3A). Among *E. coli* isolates, concordance was highest for β-lactam agents, including ampicillin (93%) and cefotaxime, ceftriaxone, and aztreonam (each 87%), largely associated with the presence of *bla*_CTX−M−15_, *bla*_OXA−1_, and *bla*_EC_. These concordant profiles were predominantly observed among isolates from tracheal aspirate and urine. Lower concordance was observed for sulfamethoxazole/trimethoprim (53%) and gentamicin (60%), with phenotypic resistance observed across multiple sample types despite the absence of corresponding resistance genes. The lowest concordance was seen for amikacin and meropenem (both 7%), each observed in a single *E. coli* urine isolate (Supplementary Table S2).

Among *K. pneumoniae* isolates, ampicillin showed the highest concordance (86%), followed by cefotaxime and ceftriaxone (71%). These cases were observed across urine, tracheal aspirate, blood, and pus swab samples. Ceftazidime and sulfamethoxazole/trimethoprim exhibited moderate concordance (both 43%), with mismatches recorded across blood, tracheal aspirate, urine, and pus samples. Regardless of species, the predominant form of discordance was phenotypic resistance in the absence of corresponding resistance genes (pheno+/geno–). A detailed breakdown of concordance categories by antibiotic is presented in Fig. 3B, with isolate-level data provided in Supplementary Table S2.

### Multilocus sequence typing (MLST)

MLST revealed substantial genetic diversity among *E. coli*, with 13 of 15 isolates assigned to seven sequence types or complexes (Table 2). High-risk lineages STc131 (*n* = 3) and ST648 (*n* = 5) dominated. STc131 and ST648b were exclusively recovered from urine and tracheal aspirate samples.

**Table 2:** Sequence type distribution and sample source among *E. coli* isolates *(n* = 15)

Sequence Type	No. of isolates	Blood	Pus swab	Tracheal aspirate	Urine
STc131	3	0	0	1	2
STc10	3	0	0	1	2
ST648	5	1	1	2	1
STc23	1	0	0	1	0
STc14	1	0	0	1	0
Not determined	2	0	0	1	1
Total	15	1	1	8	5

Among *K. pneumoniae* isolates, isolate K15 (from a tracheal aspirate) was assigned to ST405. The remaining six isolates were untypeable by MLST but were derived from diverse sample types including tracheal aspirates (*n* = 2), urine (*n* = 2), blood (*n* = 1), and pus swab (*n* = 1).

Two *E. coli* isolates and six *K. pneumoniae* isolates could not be assigned to known sequence types due to incomplete profiles at one or more housekeeping gene loci used in the MLST scheme.

### Virulence gene distribution

**Fig. 4:**
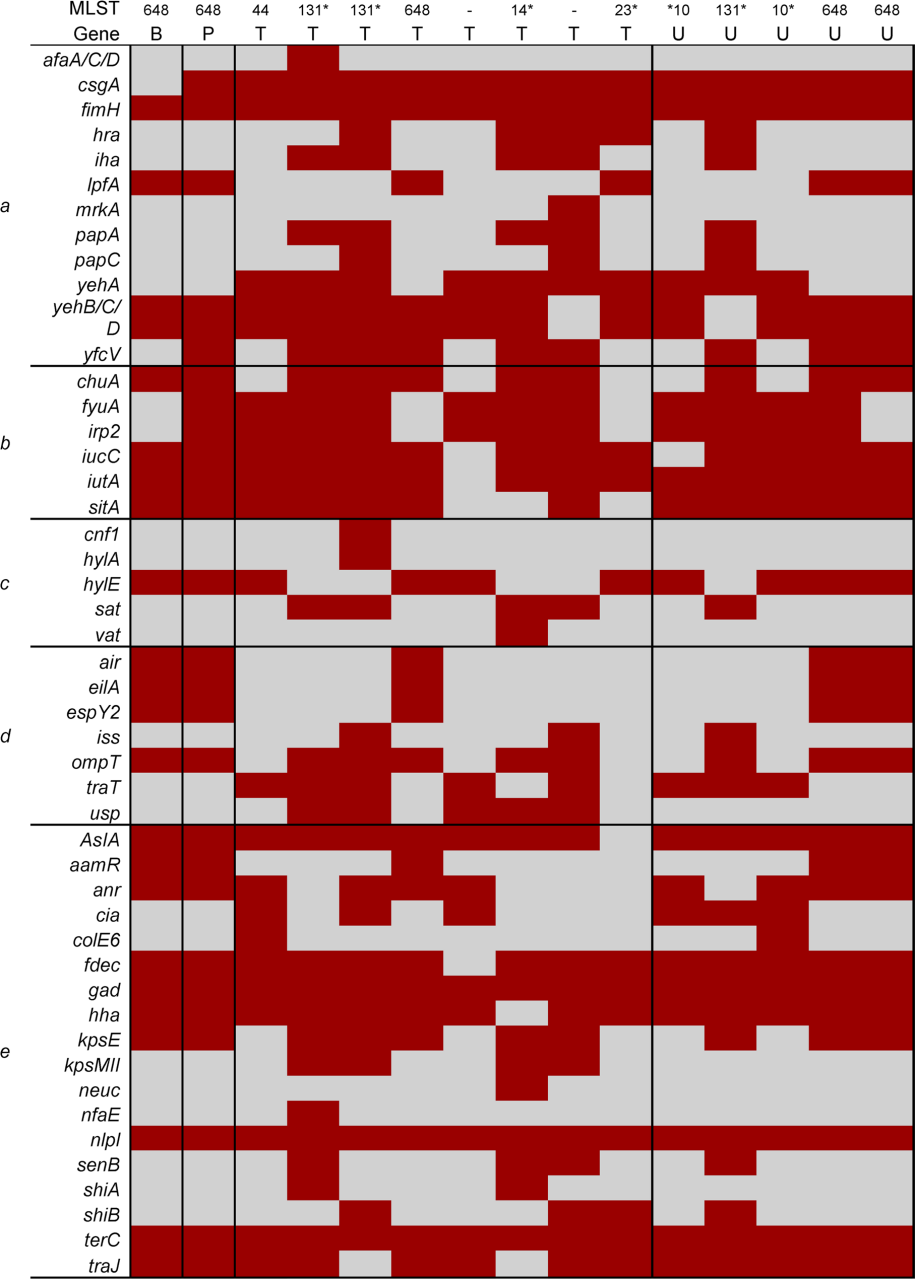
Heatmap of virulence genes distribution among *E. coli* isolates. Each column represents an individual isolate by sample type: B = blood, P = pus swab, T = tracheal aspirate, U = urine, and its sequence type, while each row represents a distinct virulence gene. Genes are grouped by functional category: **a** adhesins/fimbriae/pili, **b** iron acquisition systems, **c** toxins, **d** immune evasion factors, and **e** secretion systems and other stress/adaptation factors. Dark red indicates presence, and light grey indicates absence of the corresponding gene. *Sequence type complex (STc)

**Fig. 5:**
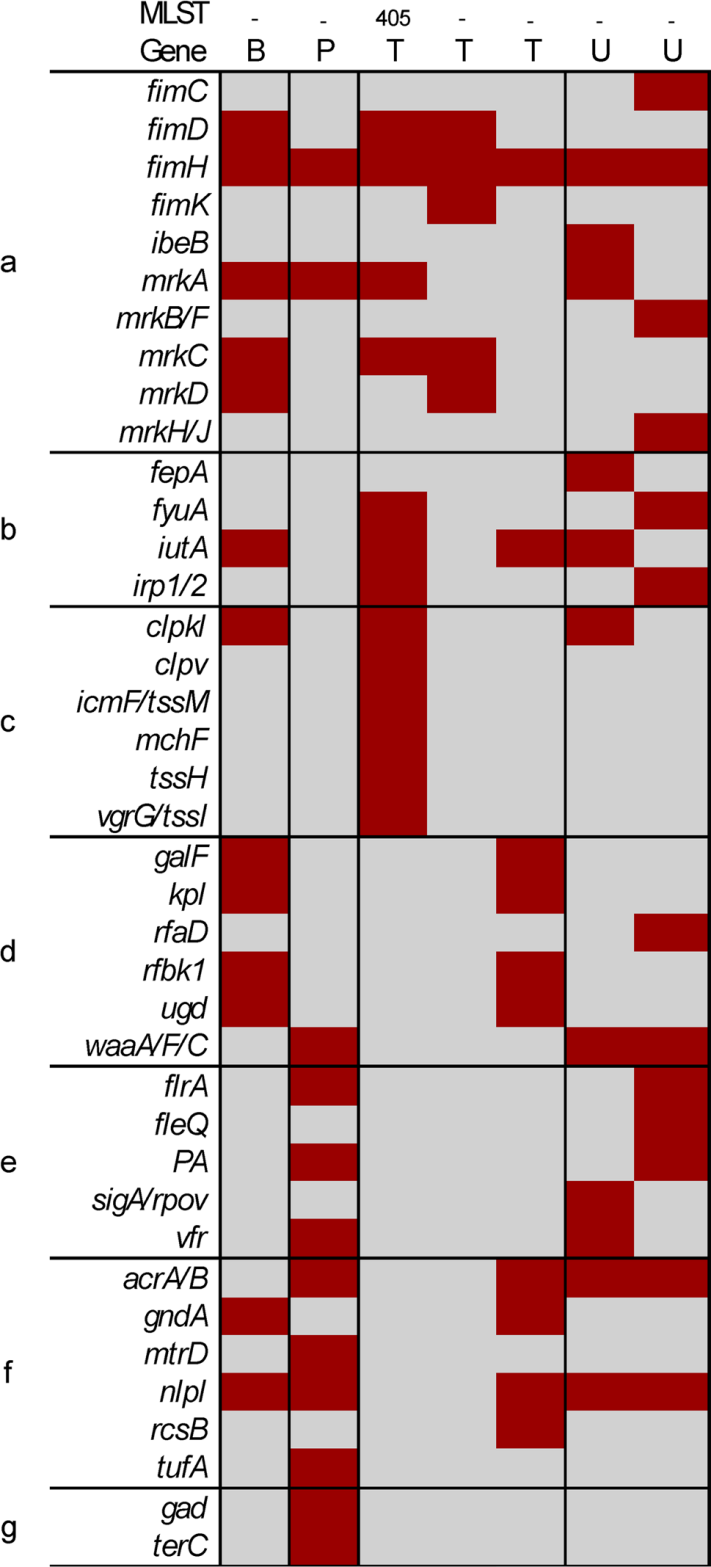
Heatmap of virulence genes distribution among *K. pneumoniae* isolates. Each column represents an individual isolate by sample type: B = blood, P = pus swab, T = tracheal aspirate, U = urine, and its sequence type, while each row represents a distinct virulence gene. Genes are grouped by functional categories: **a** fimbrial adhesins, **b** iron acquisition systems, **c** secretion machinery, **d** envelope-associated factors, **e** motility/flagella regulation, **f** Transcriptional regulation and **g** other factors. Presence of each gene is indicated in dark red, and absence in light grey

Virulence gene content varied considerably between species and by sample type (Figs. 4 and 5). Among *E. coli* isolates, those from urine (*n* = 5) and tracheal aspirates (*n* = 8) harboured a greater number and diversity of virulence determinants compared to those from blood (*n* = 1) and pus (*n* = 1). The most detected genes included *fimH* (*n* = 15, 100%), *chuA* (*n* = 8, 53%), *fyuA* (*n* = 7, 47%), *sat* (*n* = 5, 33%), *vat* (*n* = 4, 27%), and *papC* (*n* = 3, 20%). These genes were disproportionately represented among isolates belonging to STc131 and ST648. Specifically, *chuA*, *fyuA*, and *sat* were detected in 100% of STc131 isolates (*n* = 3) and in at least one ST648 isolate (Fig. 4).

*K. pneumoniae* isolates exhibited a narrower virulence gene repertoire. The ST405 isolate harboured (a) adhesins/fimbriae/pili gene fimH, (b) iron acquisition *genes fyua*,* ituA*, (c) toxins genes *Clpkl*,* clpv*,* icmF/tssM*,* mchF*,* tssH*,* vgrG/tssI*, while other isolates carried at most one or two virulence genes, typically *fimH*. No virulence gene was consistently detected across all *K. pneumoniae* isolates. There was no discernible pattern linking virulence gene presence to sample type.

### Plasmid replicon diversity

Plasmid analysis revealed a high number of replicon types among *E. coli* isolates, particularly those from tracheal aspirates and urine (Table 2). The most frequently identified replicons were *IncFII* and *IncFIB (AP001918)*, each present in 12 (80%) of 15 isolates. *IncFIA* was also common (9/15, 60%), frequently co-occurring with these replicons. Additional replicons included *col156* (*n* = 5), *col(BS512)* (*n* = 1), *IncY* (*n* = 1), and *IncFIB (H89-phage plasmid)* (*n* = 1). Two isolates (E06 and E10), all from tracheal aspirates and urine, carried six and five distinct plasmids respectively. 

Among *K. pneumoniae* isolates, five distinct plasmid types were identified. *IncFIB(K)* was the most prevalent, found in three isolates. *IncFIA (PBK30683)*,* IncHI1B (pNDM-MAR)*,* IncI1-1(α)*, and *IncR* were each identified in a single isolate. Plasmid carriage appeared more sporadic and less dense compared to *E. coli*, and no strong association with sample type was observed. The highest number of plasmids was observed in a urine sample isolate (Table 3) (Table 4).

**Table 3:** Plasmid replicons identified among the 15 *E. coli* isolates

ID	E12	E05	E03	E06	E07	E14		E19		E22	E02	E09	E10	E11	E13
MLST	648	648	44	131	131	648	-	14	-	23	10	131	10	648	648
Plasmid	B	P	T*	T*	T	T	T	T	T	T	U	U*	U	U	U
*IncFIA*	+	+	+		+	+		+		+	+		+	+	+
*IncFIB*	+	+	+	+	+	+		+	+		+	+	+	+	+
*IncFII*	+	+	+	+	+	+	+		+		+	+	+	+	+
*Incl1-1(Alpha)*			+	+	+		+	+			+	+	+		
*col156*			+	+					+			+	+		
*col (BS512)*														+	
*IncFIB*				+											
*IncY*				+											
Total	3	3	5	6	4	3	2	3	3	1	4	4	5	4	3

Each column represents an individual isolate by sample type: B = blood, P = pus swab, T = tracheal aspirate, U = urine, and its sequence type/complex. Rows indicate the presence (+) or absence (blank) of specific plasmid incompatibility groups or colicin plasmids. The total number of plasmids per isolate ranged from 1 to 6. (*) isolates E06, E07 and E 10 respectively

**Table 4:** Plasmid replicons identified among the 7 *K. pneumoniae* isolates

MLST	-	-	405	-	-	-	-
Plasmid	B	P	T	T	T	U	U
*IncFIB (K)*		+	+			+	
*IncFIA (PBK30683)*						+	
*Incl1-1 (Alpha)*				+			
*IncHI1B (pNDM-MAR)*						+	
*IncR*	+						
Total	1	1	1	1	0	3	0

Each column represents an individual isolate by sample type: B = blood, P = pus swab, T = tracheal aspirate, U = urine, and its sequence type/complex. The presence (+) or absence (blank) of specific plasmid replicons is indicated in each row. The total number of plasmids per isolate ranged from 0 to 3

Overall, Isolates carrying *IncFIA*,* IncFIB*, and *IncFII* plasmids consistently harbored ESBL genes (*bla*_CTX−M−15_, *bla*_OXA−1_) and aminoglycoside resistance genes (*aadA5*, *aac(6’)-Ib*). Isolates E06 and E10 (6 and 5 plasmids respectively) had 16 and 14 AMR genes respectively. Urine and tracheal aspirate isolates generally had more plasmids [3–6] compared to blood [3] and pus [3] isolates (Supplementary table S4).

## Discussion

The emergence of multidrug-resistant Enterobacterales, particularly *E. coli* and *K. pneumoniae*, continues to complicate clinical management, especially in intensive care settings where patients are particularly vulnerable. In this study, the widespread resistance to commonly used antibiotics—most notably ampicillin, third-generation cephalosporins, and aztreonam—reinforces the narrowing of effective treatment options. The broader resistance profile observed in *E. coli* isolates relative to *K. pneumoniae* is consistent with global surveillance trends and may reflect species-specific mechanisms for resistance acquisition and persistence [21, 22].

The detection of the ESBL gene *bla*_CTX−M−15_ in 93% of *E. coli* isolates, and 100% in *K. pneumoniae and* concordance of phenotypic resistance to beta lactam antibiotics, highlights the predominance of ESBL-mediated resistance mechanism among the isolates [23]. This demonstrates the polyclonal nature of ESBL gene dissemination across the isolates. The distribution of multidrug resistance profiles by sample type further illustrates clinically relevant patterns. These findings have direct clinical implications, as the presence of resistance phenotypes circulating in critical care environments may complicate treatment. Although the dataset is limited, the repeated identification of high-resistance profiles in these sample types merits ongoing surveillance, particularly given the potential for nosocomial transmission in ICU settings [24].

The genotypic analysis revealed notable interspecies differences. Among aminoglycoside, and quinolone resistance genes, *aac(6′)-Ib*, and *gyrA* were more prevalent in *E. coli*, whereas *oqxA* and *oqxB* were predominant among the *K. pneumoniae* isolates. The lower concordance observed between phenotype and genotype for some antibiotics, such as sulfamethoxazole/trimethoprim and gentamicin, highlights the complexity of resistance prediction. In several instances, phenotypic resistance occurred in the absence of known resistance genes, possibly reflecting alternative mechanisms such as efflux pump activation, porin mutations, or gene expression regulation [25]. While the overall agreement between genotype and phenotype was strongest for β-lactams, inconsistencies for other drug classes reinforce the importance of comprehensive resistance diagnostics that integrate both molecular and phenotypic data [26, 27].

Genetic diversity among *E. coli* isolates was substantial, with 13 of 15 strains assigned to known sequence types or complexes. Of particular interest were the repeated detections of STc131 and ST648—lineages recognized for their association with extraintestinal pathogenicity and resistance [28, 29]. These sequence types were exclusively identified in urine and tracheal aspirate samples, in line with prior studies documenting their involvement in urinary tract and lower respiratory tract infections [30, 31]. For the *K. pneumoniae* isolates, one isolate was typed as ST405, a globally distributed clone associated with hospital outbreaks and ESBL production [32]. The identification of these high-risk clones in ICU settings raises concerns about the potential for increased transmission rates and thus the urgent need for enhanced surveillance and targeted infection control measures.

Virulence profiling uncovered additional interspecies contrasts. *E. coli* isolates, particularly from urine and tracheal aspirates, exhibited extensive virulence gene content, including *fimH*, *chuA*, *fyuA*, *sat*, *vat*, and *papC*—determinants linked to adhesion, iron acquisition, and toxin production. These genes were disproportionately represented in STc131 and ST648, supporting their classification among uropathogenic and extraintestinal *E. coli* strains [33]. In contrast, *K. pneumoniae* displayed a narrower and more heterogeneous virulence gene profile, with detection of *fimH* in all seven isolates. The absence of consistent virulence markers across *K. pneumoniae* isolates may reflect differing ecological adaptations or incomplete virulence genotyping in the current dataset [34].

Plasmid replicon analysis further highlighted the genomic divergence between species. *E. coli* isolates carried a wider range of plasmids, with *IncFII* and *IncFIB (AP001918)* detected in over 80% of strains. These replicons are frequently implicated in the carriage of both resistance and virulence genes, suggesting that plasmid-mediated gene transfer plays a central role in shaping *E. coli*’s pathogenic potential in this setting [35]. *K. pneumoniae* isolates harbored fewer replicon types, which were more sporadically distributed, with *IncFIB(K)* as the most common. Several isolates carried as many as six distinct plasmids. Isolates carrying *IncFIA*,* IncFIB*, and IncFII plasmids consistently harbored core ESBL genes (*bla*_CTX−M−15_, *bla*_OXA−1_) and aminoglycoside resistance genes (*aadA5*,* aac(6’)-Ib*). The high plasmid burden correlates with extensive resistance and indicates a high capacity for horizontal gene exchange.

This study is not without limitations. The relatively small sample size and focus on a single healthcare facility restrict the generalizability of the findings. Furthermore, the absence of clinical outcome data and gene expression profiling limits interpretation of the functional impact of the identified resistance and virulence determinants. Nevertheless, the high-resolution genomic data presented here offers an important snapshot of the molecular epidemiology of multidrug-resistant *E. coli* and *K. pneumoniae* in a high-risk clinical context.

## Conclusion

This study highlights the co-occurrence of high-risk sequence types and extensive resistance profiles, revealing how genetic background, virulence traits, and plasmid repertoires collectively shape the pathogenic potential of multidrug-resistant Enterobacterales. The predominance of bla_CTX−M−15_ highlights its critical role in limiting therapeutic options, particularly in intensive care settings where vulnerable patients face heightened risks. Notably, *E. coli* isolates showed broader resistance, virulence, and plasmid diversity, consistent with their central role in global AMR dissemination. Immediate action is needed to implement strengthened surveillance systems, molecular diagnostics, and tailored stewardship policies to contain the spread of these pathogens and safeguard patient care in critical settings. 

## Supplementary Information

Below is the link to the electronic supplementary material.

Supplementary Material 1.

Supplementary Material 2.

Supplementary Material 3.

Supplementary Material 4.

## Data Availability

All data generated or analyzed during this study are included in this published article [and its supplementary information files]. The datasets generated and/or analysed during the current study are available in Figshare. Phenotypic and genomic data for Extended Spectrum Beta Lactamase cephalosporin resistant bacterial isolates from ICU at a referral hospital in Kenya: Figshare. Dataset. [https://doi.org/10.6084/m9.figshare.29291150]. The Whole-genome sequences are deposited in NCBI BioProject ID PRJNA1306555.

## Abbreviations

AMR: Antimicrobial Resistance
AST: Antimicrobial Susceptibility Testing
ESBL: Extended-Spectrum Beta-Lactamase
ICU: Intensive Care Unit
KNH: Kenyatta National Hospital
MLST: Multilocus Sequence Typing
ONT: Oxford Nanopore Technology
STc: Sequence Type Complex
WGS: Whole Genome Sequencing
