# Supplementary material for: Phenotypic and genomic profiling of multidrug-resistant Escherichia coli and Klebsiella pneumoniae isolated from Intensive Care Unit patients in Kenya
Source: BMC Microbiol. 2026 Mar 23;26:419. doi: 10.1186/s12866-026-04880-5 (PMC13130711; doi:10.1186/s12866-026-04880-5)
Supplement: Supplementary file 1 — Supplementary Material 1. [file 12866_2026_4880_MOESM1_ESM.docx]

**Supplementary Table 1. AMR genes identified among the *E. coli* and *K. pneumoniae Isolates.***

| Encoded resistance | Gene detected | *E. coli*  *(N=15)* | | | *K. pneumoniae (N=7)* |  |
| --- | --- | --- | --- | --- | --- | --- |
|  |  | n (%) | | | n (%) |  |
| Aminoglycosides | *aac(3’)-la* | |  | 1(7) | 2(29) |  |
|  | *aac(6’)lb-cr* | |  | 12(80) | 0 |  |
|  | *aadA* | |  | 13(87) | 2(29) |  |
|  | *aph(3”)-lb* | |  | 6(40) | 7(100) |  |
|  | *aph(3”)-lld* | |  |  | 2(29) |  |
|  | *aph(6)-ld* | |  | 3(20) | 0 |  |
| Beta-Lactams | *blaCMY* | |  | 1(7) | 0 |  |
|  | *bla CTXM-15* | |  | 14(93) | 7(100) |  |
|  | *blaEC* | |  | 11(73) | 0 |  |
|  | *blaOXA-1* | |  | 13(87) | 0 |  |
|  | *blaOXA-534* | |  | 3(20) | 0 |  |
|  | *blaSHV-17* | |  | 2(13) | 7(100) |  |
|  | *blaTEM-1* | |  | 5(33) | 5(71) |  |
| Phenicol | *catA* | |  | 0 | 1(14) |  |
|  | *catB* | |  | 8(53) | 0 |  |
| Quinolones | *gyrA* | |  | 13(87) | 0 |  |
|  | *oqxA* | |  | 3(20) | 7(100) |  |
|  | *oqxB* | |  |  | 5(71) |  |
|  | *qnrB* | |  | 0 | 4(57) |  |
| Sulfonamides | *dfrA* | |  | 9(60) | 6(86) |  |
|  | *sul1* | |  | 13(87) | 2(29) |  |
|  | *sul2* | |  | 4(27) | 7(100) |  |
| Tetracyclines | *tetA* | |  | 3(20) | 2(29) |  |
|  | *tetB* | |  | 10(67) | 2(29) |  |
|  | *tetD* | |  | 2(13) | 1(14) |  |
| Fosfomycin | *fosA* | |  | 4(27) | 5(71) |  |
