# Supplementary material for: Phenotypic and genomic profiling of multidrug-resistant Escherichia coli and Klebsiella pneumoniae isolated from Intensive Care Unit patients in Kenya
Source: BMC Microbiol. 2026 Mar 23;26:419. doi: 10.1186/s12866-026-04880-5 (PMC13130711; doi:10.1186/s12866-026-04880-5)
Supplement: Supplementary file 2 — Supplementary Material 2. [file 12866_2026_4880_MOESM2_ESM.docx]

**Supplementary Table S2. Isolate-level concordance between phenotypic antimicrobial resistance and the presence of corresponding resistance genes in *Escherichia coli* (n = 15) and *Klebsiella pneumoniae* (n = 7).** This table presents a detailed concordance analysis for each isolate across 18 antibiotics. For each isolate–antibiotic combination, phenotypic resistance was matched against the presence of known resistance genes associated with that drug class. Concordance was classified as: Concordant (+/+) if both resistance and gene were present; Concordant (–/–) if neither was present; Pheno+/Geno– if phenotypically resistant but lacking a known gene; or Pheno–/Geno+ if phenotypically susceptible despite harbouring resistance genes. AMP, ampicillin; AMC, amoxicillin/clavulanate; AMK, amikacin; ATM, aztreonam; CAZ, ceftazidime; CEZ, cefazolin; CIP, ciprofloxacin; CRO, ceftriaxone; CTX, cefotaxime; CXM, cefuroxime; CXMA, cefuroxime/axetil; FEP, cefepime; GEN, gentamicin; MEM, meropenem; SAM, ampicillin/sulbactam; SXT, sulfamethoxazole/ trimethoprim; TZP, piperacillin/tazobactam; FT: Nitrofurantoin.

| **Isolate ID** | **Sample type** | **Antibiotic** | **Phenotypic resistance** | **Resistance gene detected** | **Classification** |
| --- | --- | --- | --- | --- | --- |
| E. coli_02 | Urine | AMP | Resistant | Yes | Concordant (+/+) |
| E. coli_02 | Urine | AMC | Susceptible | Yes | Pheno-/Geno+ |
| E. coli_02 | Urine | AMK | Susceptible | Yes | Pheno-/Geno+ |
| E. coli_02 | Urine | ATM | Resistant | Yes | Concordant (+/+) |
| E. coli_02 | Urine | CAZ | Resistant | Yes | Concordant (+/+) |
| E. coli_02 | Urine | CEZ | Resistant | Yes | Concordant (+/+) |
| E. coli_02 | Urine | CIP | Resistant | Yes | Concordant (+/+) |
| E. coli_02 | Urine | CRO | Resistant | Yes | Concordant (+/+) |
| E. coli_02 | Urine | CTX | Resistant | Yes | Concordant (+/+) |
| E. coli_02 | Urine | CXM | Resistant | Yes | Concordant (+/+) |
| E. coli_02 | Urine | CXMA | Resistant | Yes | Concordant (+/+) |
| E. coli_02 | Urine | FEP | Susceptible | Yes | Pheno-/Geno+ |
| E. coli_02 | Urine | GEN | Susceptible | Yes | Pheno-/Geno+ |
| E. coli_02 | Urine | MEM | Susceptible | No | Concordant (-/-) |
| E. coli_02 | Urine | SAM | Resistant | Yes | Concordant (+/+) |
| E. coli_02 | Urine | SXT | Resistant | Yes | Concordant (+/+) |
| E. coli_02 | Urine | TZP | Susceptible | Yes | Pheno-/Geno+ |
| E. coli_02 | Urine | FT | Susceptible | No | Concordant (-/-) |
| E. coli_03 | Tracheal aspirate | AMP | Resistant | Yes | Concordant (+/+) |
| E. coli_03 | Tracheal aspirate | AMC | Resistant | Yes | Concordant (+/+) |
| E. coli_03 | Tracheal aspirate | AMK | Susceptible | Yes | Pheno-/Geno+ |
| E. coli_03 | Tracheal aspirate | ATM | Resistant | Yes | Concordant (+/+) |
| E. coli_03 | Tracheal aspirate | CAZ | Resistant | Yes | Concordant (+/+) |
| E. coli_03 | Tracheal aspirate | CEZ | Resistant | Yes | Concordant (+/+) |
| E. coli_03 | Tracheal aspirate | CIP | Resistant | Yes | Concordant (+/+) |
| E. coli_03 | Tracheal aspirate | CRO | Resistant | Yes | Concordant (+/+) |
| E. coli_03 | Tracheal aspirate | CTX | Resistant | Yes | Concordant (+/+) |
| E. coli_03 | Tracheal aspirate | CXM | Resistant | Yes | Concordant (+/+) |
| E. coli_03 | Tracheal aspirate | CXMA | Resistant | Yes | Concordant (+/+) |
| E. coli_03 | Tracheal aspirate | FEP | Resistant | Yes | Concordant (+/+) |
| E. coli_03 | Tracheal aspirate | GEN | Resistant | Yes | Concordant (+/+) |
| E. coli_03 | Tracheal aspirate | MEM | Susceptible | No | Concordant (-/-) |
| E. coli_03 | Tracheal aspirate | SAM | Resistant | Yes | Concordant (+/+) |
| E. coli_03 | Tracheal aspirate | SXT | Resistant | Yes | Concordant (+/+) |
| E. coli_03 | Tracheal aspirate | TZP | Resistant | Yes | Concordant (+/+) |
| E. coli_03 | Tracheal aspirate | FT | Susceptible | No | Concordant (-/-) |
| E. coli_05 | Pus swab | AMP | Resistant | Yes | Concordant (+/+) |
| E. coli_05 | Pus swab | AMC | Susceptible | Yes | Pheno-/Geno+ |
| E. coli_05 | Pus swab | AMK | Susceptible | Yes | Pheno-/Geno+ |
| E. coli_05 | Pus swab | ATM | Resistant | Yes | Concordant (+/+) |
| E. coli_05 | Pus swab | CAZ | Resistant | Yes | Concordant (+/+) |
| E. coli_05 | Pus swab | CEZ | Resistant | Yes | Concordant (+/+) |
| E. coli_05 | Pus swab | CIP | Resistant | Yes | Concordant (+/+) |
| E. coli_05 | Pus swab | CRO | Resistant | Yes | Concordant (+/+) |
| E. coli_05 | Pus swab | CTX | Resistant | Yes | Concordant (+/+) |
| E. coli_05 | Pus swab | CXM | Resistant | Yes | Concordant (+/+) |
| E. coli_05 | Pus swab | CXMA | Resistant | Yes | Concordant (+/+) |
| E. coli_05 | Pus swab | FEP | Susceptible | Yes | Pheno-/Geno+ |
| E. coli_05 | Pus swab | GEN | Susceptible | Yes | Pheno-/Geno+ |
| E. coli_05 | Pus swab | MEM | Susceptible | No | Concordant (-/-) |
| E. coli_05 | Pus swab | SAM | Resistant | Yes | Concordant (+/+) |
| E. coli_05 | Pus swab | SXT | Resistant | Yes | Concordant (+/+) |
| E. coli_05 | Pus swab | TZP | Susceptible | Yes | Pheno-/Geno+ |
| E. coli_05 | Pus swab | FT | Susceptible | No | Concordant (-/-) |
| E. coli_06 | Tracheal aspirate | AMP | Resistant | Yes | Concordant (+/+) |
| E. coli_06 | Tracheal aspirate | AMC | Susceptible | Yes | Pheno-/Geno+ |
| E. coli_06 | Tracheal aspirate | AMK | Susceptible | Yes | Pheno-/Geno+ |
| E. coli_06 | Tracheal aspirate | ATM | Resistant | Yes | Concordant (+/+) |
| E. coli_06 | Tracheal aspirate | CAZ | Resistant | Yes | Concordant (+/+) |
| E. coli_06 | Tracheal aspirate | CEZ | Resistant | Yes | Concordant (+/+) |
| E. coli_06 | Tracheal aspirate | CIP | Susceptible | No | Concordant (-/-) |
| E. coli_06 | Tracheal aspirate | CRO | Resistant | Yes | Concordant (+/+) |
| E. coli_06 | Tracheal aspirate | CTX | Resistant | Yes | Concordant (+/+) |
| E. coli_06 | Tracheal aspirate | CXM | Resistant | Yes | Concordant (+/+) |
| E. coli_06 | Tracheal aspirate | CXMA | Resistant | Yes | Concordant (+/+) |
| E. coli_06 | Tracheal aspirate | FEP | Susceptible | Yes | Pheno-/Geno+ |
| E. coli_06 | Tracheal aspirate | GEN | Resistant | Yes | Concordant (+/+) |
| E. coli_06 | Tracheal aspirate | MEM | Susceptible | No | Concordant (-/-) |
| E. coli_06 | Tracheal aspirate | SAM | Resistant | Yes | Concordant (+/+) |
| E. coli_06 | Tracheal aspirate | SXT | Resistant | Yes | Concordant (+/+) |
| E. coli_06 | Tracheal aspirate | TZP | Susceptible | No | Concordant (-/-) |
| E. coli_06 | Tracheal aspirate | FT | Susceptible | Yes | Concordant (-/-) |
| E. coli_07 | Tracheal aspirate | AMP | Resistant | Yes | Concordant (+/+) |
| E. coli_07 | Tracheal aspirate | AMC | Susceptible | Yes | Pheno-/Geno+ |
| E. coli_07 | Tracheal aspirate | AMK | Susceptible | Yes | Pheno-/Geno+ |
| E. coli_07 | Tracheal aspirate | ATM | Resistant | Yes | Concordant (+/+) |
| E. coli_07 | Tracheal aspirate | CAZ | Resistant | Yes | Concordant (+/+) |
| E. coli_07 | Tracheal aspirate | CEZ | Resistant | Yes | Concordant (+/+) |
| E. coli_07 | Tracheal aspirate | CIP | Resistant | Yes | Concordant (+/+) |
| E. coli_07 | Tracheal aspirate | CRO | Resistant | Yes | Concordant (+/+) |
| E. coli_07 | Tracheal aspirate | CTX | Resistant | Yes | Concordant (+/+) |
| E. coli_07 | Tracheal aspirate | CXM | Resistant | Yes | Concordant (+/+) |
| E. coli_07 | Tracheal aspirate | CXMA | Resistant | Yes | Concordant (+/+) |
| E. coli_07 | Tracheal aspirate | FEP | Susceptible | Yes | Pheno-/Geno+ |
| E. coli_07 | Tracheal aspirate | GEN | Resistant | Yes | Concordant (+/+) |
| E. coli_07 | Tracheal aspirate | MEM | Susceptible | No | Concordant (-/-) |
| E. coli_07 | Tracheal aspirate | SAM | Resistant | Yes | Concordant (+/+) |
| E. coli_07 | Tracheal aspirate | SXT | Resistant | Yes | Concordant (+/+) |
| E. coli_07 | Tracheal aspirate | TZP | Susceptible | Yes | Pheno-/Geno+ |
| E. coli_07 | Tracheal aspirate | FT | Susceptible | No | Concordant (-/-) |
| E. coli_09 | Urine | AMP | Resistant | Yes | Concordant (+/+) |
| E. coli_09 | Urine | AMC | Susceptible | Yes | Pheno-/Geno+ |
| E. coli_09 | Urine | AMK | Susceptible | Yes | Pheno-/Geno+ |
| E. coli_09 | Urine | ATM | Resistant | Yes | Concordant (+/+) |
| E. coli_09 | Urine | CAZ | Resistant | Yes | Concordant (+/+) |
| E. coli_09 | Urine | CEZ | Resistant | Yes | Concordant (+/+) |
| E. coli_09 | Urine | CIP | Resistant | Yes | Concordant (+/+) |
| E. coli_09 | Urine | CRO | Resistant | Yes | Concordant (+/+) |
| E. coli_09 | Urine | CTX | Resistant | Yes | Concordant (+/+) |
| E. coli_09 | Urine | CXM | Resistant | Yes | Concordant (+/+) |
| E. coli_09 | Urine | CXMA | Resistant | Yes | Concordant (+/+) |
| E. coli_09 | Urine | FEP | Susceptible | Yes | Pheno-/Geno+ |
| E. coli_09 | Urine | GEN | Resistant | Yes | Concordant (+/+) |
| E. coli_09 | Urine | MEM | Susceptible | No | Concordant (-/-) |
| E. coli_09 | Urine | SAM | Resistant | Yes | Concordant (+/+) |
| E. coli_09 | Urine | SXT | Resistant | Yes | Concordant (+/+) |
| E. coli_09 | Urine | TZP | Susceptible | Yes | Pheno-/Geno+ |
| E. coli_09 | Urine | FT | Susceptible | No | Concordant (-/-) |
| E. coli_10 | Urine | AMP | Resistant | Yes | Concordant (+/+) |
| E. coli_10 | Urine | AMC | Susceptible | Yes | Pheno-/Geno+ |
| E. coli_10 | Urine | AMK | Susceptible | Yes | Pheno-/Geno+ |
| E. coli_10 | Urine | ATM | Resistant | Yes | Concordant (+/+) |
| E. coli_10 | Urine | CAZ | Resistant | Yes | Concordant (+/+) |
| E. coli_10 | Urine | CEZ | Resistant | Yes | Concordant (+/+) |
| E. coli_10 | Urine | CIP | Resistant | Yes | Concordant (+/+) |
| E. coli_10 | Urine | CRO | Resistant | Yes | Concordant (+/+) |
| E. coli_10 | Urine | CTX | Resistant | Yes | Concordant (+/+) |
| E. coli_10 | Urine | CXM | Resistant | Yes | Concordant (+/+) |
| E. coli_10 | Urine | CXMA | Resistant | Yes | Concordant (+/+) |
| E. coli_10 | Urine | FEP | Susceptible | Yes | Pheno-/Geno+ |
| E. coli_10 | Urine | GEN | Susceptible | Yes | Pheno-/Geno+ |
| E. coli_10 | Urine | MEM | Susceptible | No | Concordant (-/-) |
| E. coli_10 | Urine | SAM | Resistant | Yes | Concordant (+/+) |
| E. coli_10 | Urine | SXT | Resistant | Yes | Concordant (+/+) |
| E. coli_10 | Urine | TZP | Susceptible | Yes | Pheno-/Geno+ |
| E. coli_10 | Urine | FT | Susceptible | No | Concordant (-/-) |
| E. coli_11 | Urine | AMP | Resistant | Yes | Concordant (+/+) |
| E. coli_11 | Urine | AMC | Susceptible | Yes | Pheno-/Geno+ |
| E. coli_11 | Urine | AMK | Susceptible | Yes | Pheno-/Geno+ |
| E. coli_11 | Urine | ATM | Resistant | Yes | Concordant (+/+) |
| E. coli_11 | Urine | CAZ | Resistant | Yes | Concordant (+/+) |
| E. coli_11 | Urine | CEZ | Resistant | Yes | Concordant (+/+) |
| E. coli_11 | Urine | CIP | Resistant | Yes | Concordant (+/+) |
| E. coli_11 | Urine | CRO | Resistant | Yes | Concordant (+/+) |
| E. coli_11 | Urine | CTX | Resistant | Yes | Concordant (+/+) |
| E. coli_11 | Urine | CXM | Resistant | Yes | Concordant (+/+) |
| E. coli_11 | Urine | CXMA | Resistant | Yes | Concordant (+/+) |
| E. coli_11 | Urine | FEP | Susceptible | Yes | Pheno-/Geno+ |
| E. coli_11 | Urine | GEN | Susceptible | Yes | Pheno-/Geno+ |
| E. coli_11 | Urine | MEM | Susceptible | No | Concordant (-/-) |
| E. coli_11 | Urine | SAM | Resistant | Yes | Concordant (+/+) |
| E. coli_11 | Urine | SXT | Resistant | Yes | Concordant (+/+) |
| E. coli_11 | Urine | TZP | Susceptible | Yes | Pheno-/Geno+ |
| E. coli_11 | Urine | FT | Susceptible | No | Concordant (-/-) |
| E. coli_12 | Blood | AMP | Resistant | Yes | Concordant (+/+) |
| E. coli_12 | Blood | AMC | Resistant | Yes | Concordant (+/+) |
| E. coli_12 | Blood | AMK | Susceptible | Yes | Pheno-/Geno+ |
| E. coli_12 | Blood | ATM | Resistant | Yes | Concordant (+/+) |
| E. coli_12 | Blood | CAZ | Resistant | Yes | Concordant (+/+) |
| E. coli_12 | Blood | CEZ | Resistant | Yes | Concordant (+/+) |
| E. coli_12 | Blood | CIP | Susceptible | Yes | Pheno-/Geno+ |
| E. coli_12 | Blood | CRO | Resistant | Yes | Concordant (+/+) |
| E. coli_12 | Blood | CTX | Resistant | Yes | Concordant (+/+) |
| E. coli_12 | Blood | CXM | Resistant | Yes | Concordant (+/+) |
| E. coli_12 | Blood | CXMA | Resistant | Yes | Concordant (+/+) |
| E. coli_12 | Blood | FEP | Susceptible | Yes | Pheno-/Geno+ |
| E. coli_12 | Blood | GEN | Susceptible | Yes | Pheno-/Geno+ |
| E. coli_12 | Blood | MEM | Susceptible | No | Concordant (-/-) |
| E. coli_12 | Blood | SAM | Resistant | Yes | Concordant (+/+) |
| E. coli_12 | Blood | SXT | Susceptible | Yes | Pheno-/Geno+ |
| E. coli_12 | Blood | TZP | Resistant | Yes | Concordant (+/+) |
| E. coli_12 | Blood | FT | Resistant | Yes | Pheno+/Geno- |
| E. coli_13 | Urine | AMP | Resistant | Yes | Concordant (+/+) |
| E. coli_13 | Urine | AMC | Resistant | Yes | Concordant (+/+) |
| E. coli_13 | Urine | AMK | Resistant | Yes | Concordant (+/+) |
| E. coli_13 | Urine | ATM | Resistant | Yes | Concordant (+/+) |
| E. coli_13 | Urine | CAZ | Resistant | Yes | Concordant (+/+) |
| E. coli_13 | Urine | CEZ | Resistant | Yes | Concordant (+/+) |
| E. coli_13 | Urine | CIP | Resistant | Yes | Concordant (+/+) |
| E. coli_13 | Urine | CRO | Resistant | Yes | Concordant (+/+) |
| E. coli_13 | Urine | CTX | Resistant | Yes | Concordant (+/+) |
| E. coli_13 | Urine | CXM | Resistant | Yes | Concordant (+/+) |
| E. coli_13 | Urine | CXMA | Resistant | Yes | Concordant (+/+) |
| E. coli_13 | Urine | FEP | Resistant | Yes | Concordant (+/+) |
| E. coli_13 | Urine | GEN | Resistant | Yes | Concordant (+/+) |
| E. coli_13 | Urine | MEM | Resistant | No | Pheno+/Geno- |
| E. coli_13 | Urine | SAM | Resistant | Yes | Concordant (+/+) |
| E. coli_13 | Urine | SXT | Resistant | Yes | Concordant (+/+) |
| E. coli_13 | Urine | TZP | Resistant | Yes | Concordant (+/+) |
| E. coli_13 | Urine | FT | Resistant | Yes | Pheno+/Geno- |
| E. coli_14 | Tracheal aspirate | AMP | Resistant | Yes | Concordant (+/+) |
| E. coli_14 | Tracheal aspirate | AMC | Susceptible | Yes | Pheno-/Geno+ |
| E. coli_14 | Tracheal aspirate | AMK | Susceptible | Yes | Pheno-/Geno+ |
| E. coli_14 | Tracheal aspirate | ATM | Resistant | Yes | Concordant (+/+) |
| E. coli_14 | Tracheal aspirate | CAZ | Resistant | Yes | Concordant (+/+) |
| E. coli_14 | Tracheal aspirate | CEZ | Resistant | Yes | Concordant (+/+) |
| E. coli_14 | Tracheal aspirate | CIP | Resistant | Yes | Concordant (+/+) |
| E. coli_14 | Tracheal aspirate | CRO | Resistant | Yes | Concordant (+/+) |
| E. coli_14 | Tracheal aspirate | CTX | Resistant | Yes | Concordant (+/+) |
| E. coli_14 | Tracheal aspirate | CXM | Resistant | Yes | Concordant (+/+) |
| E. coli_14 | Tracheal aspirate | CXMA | Resistant | Yes | Concordant (+/+) |
| E. coli_14 | Tracheal aspirate | FEP | Susceptible | Yes | Pheno-/Geno+ |
| E. coli_14 | Tracheal aspirate | GEN | Susceptible | Yes | Pheno-/Geno+ |
| E. coli_14 | Tracheal aspirate | MEM | Susceptible | No | Concordant (-/-) |
| E. coli_14 | Tracheal aspirate | SAM | Resistant | Yes | Concordant (+/+) |
| E. coli_14 | Tracheal aspirate | SXT | Resistant | Yes | Concordant (+/+) |
| E. coli_14 | Tracheal aspirate | TZP | Susceptible | Yes | Pheno-/Geno+ |
| E. coli_14 | Tracheal aspirate | FT | Susceptible | No | Concordant (-/-) |
| E. coli_16 | Tracheal aspirate | AMP | Resistant | Yes | Concordant (+/+) |
| E. coli_16 | Tracheal aspirate | AMC | Susceptible | Yes | Pheno-/Geno+ |
| E. coli_16 | Tracheal aspirate | AMK | Susceptible | Yes | Pheno-/Geno+ |
| E. coli_16 | Tracheal aspirate | ATM | Susceptible | Yes | Pheno-/Geno+ |
| E. coli_16 | Tracheal aspirate | CAZ | Susceptible | Yes | Pheno-/Geno+ |
| E. coli_16 | Tracheal aspirate | CEZ | Resistant | Yes | Concordant (+/+) |
| E. coli_16 | Tracheal aspirate | CIP | Resistant | Yes | Concordant (+/+) |
| E. coli_16 | Tracheal aspirate | CRO | Resistant | Yes | Concordant (+/+) |
| E. coli_16 | Tracheal aspirate | CTX | Resistant | Yes | Concordant (+/+) |
| E. coli_16 | Tracheal aspirate | CXM | Resistant | Yes | Concordant (+/+) |
| E. coli_16 | Tracheal aspirate | CXMA | Resistant | Yes | Concordant (+/+) |
| E. coli_16 | Tracheal aspirate | FEP | Susceptible | Yes | Pheno-/Geno+ |
| E. coli_16 | Tracheal aspirate | GEN | Susceptible | Yes | Pheno-/Geno+ |
| E. coli_16 | Tracheal aspirate | MEM | Susceptible | No | Concordant (-/-) |
| E. coli_16 | Tracheal aspirate | SAM | Resistant | Yes | Concordant (+/+) |
| E. coli_16 | Tracheal aspirate | SXT | Resistant | Yes | Concordant (+/+) |
| E. coli_16 | Tracheal aspirate | TZP | Susceptible | Yes | Pheno-/Geno+ |
| E. coli_16 | Tracheal aspirate | FT | Susceptible | No | Concordant (-/-) |
| E. coli_19 | Tracheal aspirate | AMP | Resistant | Yes | Concordant (+/+) |
| E. coli_19 | Tracheal aspirate | AMC | Susceptible | Yes | Pheno-/Geno+ |
| E. coli_19 | Tracheal aspirate | AMK | Susceptible | No | Concordant (-/-) |
| E. coli_19 | Tracheal aspirate | ATM | Resistant | Yes | Concordant (+/+) |
| E. coli_19 | Tracheal aspirate | CAZ | Resistant | Yes | Concordant (+/+) |
| E. coli_19 | Tracheal aspirate | CEZ | Resistant | Yes | Concordant (+/+) |
| E. coli_19 | Tracheal aspirate | CIP | Resistant | No | Pheno+/Geno- |
| E. coli_19 | Tracheal aspirate | CRO | Resistant | Yes | Concordant (+/+) |
| E. coli_19 | Tracheal aspirate | CTX | Resistant | Yes | Concordant (+/+) |
| E. coli_19 | Tracheal aspirate | CXM | Resistant | Yes | Concordant (+/+) |
| E. coli_19 | Tracheal aspirate | CXMA | Resistant | Yes | Concordant (+/+) |
| E. coli_19 | Tracheal aspirate | FEP | Resistant | Yes | Concordant (+/+) |
| E. coli_19 | Tracheal aspirate | GEN | Susceptible | No | Concordant (-/-) |
| E. coli_19 | Tracheal aspirate | MEM | Susceptible | No | Concordant (-/-) |
| E. coli_19 | Tracheal aspirate | SAM | Susceptible | Yes | Pheno-/Geno+ |
| E. coli_19 | Tracheal aspirate | SXT | Resistant | Yes | Concordant (+/+) |
| E. coli_19 | Tracheal aspirate | TZP | Susceptible | Yes | Pheno-/Geno+ |
| E. coli_19 | Tracheal aspirate | FT | Susceptible | No | Concordant (-/-) |
| E. coli_20 | Tracheal aspirate | AMP | Resistant | Yes | Concordant (+/+) |
| E. coli_20 | Tracheal aspirate | AMC | Susceptible | Yes | Pheno-/Geno+ |
| E. coli_20 | Tracheal aspirate | AMK | Susceptible | Yes | Pheno-/Geno+ |
| E. coli_20 | Tracheal aspirate | ATM | Resistant | Yes | Concordant (+/+) |
| E. coli_20 | Tracheal aspirate | CAZ | Resistant | Yes | Concordant (+/+) |
| E. coli_20 | Tracheal aspirate | CEZ | Resistant | Yes | Concordant (+/+) |
| E. coli_20 | Tracheal aspirate | CIP | Resistant | Yes | Concordant (+/+) |
| E. coli_20 | Tracheal aspirate | CRO | Resistant | Yes | Concordant (+/+) |
| E. coli_20 | Tracheal aspirate | CTX | Resistant | Yes | Concordant (+/+) |
| E. coli_20 | Tracheal aspirate | CXM | Resistant | Yes | Concordant (+/+) |
| E. coli_20 | Tracheal aspirate | CXMA | Resistant | Yes | Concordant (+/+) |
| E. coli_20 | Tracheal aspirate | FEP | Susceptible | Yes | Pheno-/Geno+ |
| E. coli_20 | Tracheal aspirate | GEN | Resistant | Yes | Concordant (+/+) |
| E. coli_20 | Tracheal aspirate | MEM | Susceptible | No | Concordant (-/-) |
| E. coli_20 | Tracheal aspirate | SAM | Resistant | Yes | Concordant (+/+) |
| E. coli_20 | Tracheal aspirate | SXT | Resistant | Yes | Concordant (+/+) |
| E. coli_20 | Tracheal aspirate | TZP | Susceptible | Yes | Pheno-/Geno+ |
| E. coli_20 | Tracheal aspirate | FT | Susceptible | No | Concordant (-/-) |
| E. coli_22 | Tracheal aspirate | AMP | Resistant | No | Pheno+/Geno- |
| E. coli_22 | Tracheal aspirate | AMC | Resistant | No | Pheno+/Geno- |
| E. coli_22 | Tracheal aspirate | AMK | Susceptible | No | Concordant (-/-) |
| E. coli_22 | Tracheal aspirate | ATM | Resistant | No | Pheno+/Geno- |
| E. coli_22 | Tracheal aspirate | CAZ | Resistant | No | Pheno+/Geno- |
| E. coli_22 | Tracheal aspirate | CEZ | Resistant | No | Pheno+/Geno- |
| E. coli_22 | Tracheal aspirate | CIP | Resistant | No | Pheno+/Geno- |
| E. coli_22 | Tracheal aspirate | CRO | Resistant | No | Pheno+/Geno- |
| E. coli_22 | Tracheal aspirate | CTX | Resistant | No | Pheno+/Geno- |
| E. coli_22 | Tracheal aspirate | CXM | Resistant | No | Pheno+/Geno- |
| E. coli_22 | Tracheal aspirate | CXMA | Resistant | No | Pheno+/Geno- |
| E. coli_22 | Tracheal aspirate | FEP | Resistant | No | Pheno+/Geno- |
| E. coli_22 | Tracheal aspirate | GEN | Resistant | No | Pheno+/Geno- |
| E. coli_22 | Tracheal aspirate | MEM | Susceptible | No | Concordant (-/-) |
| E. coli_22 | Tracheal aspirate | SAM | Resistant | No | Pheno+/Geno- |
| E. coli_22 | Tracheal aspirate | SXT | Resistant | No | Pheno+/Geno- |
| E. coli_22 | Tracheal aspirate | TZP | Resistant | No | Pheno+/Geno- |
| E. coli_22 | Tracheal aspirate | FT | Susceptible | No | Concordant (-/-) |
| Klebsiella_04 | Tracheal aspirate | AMP | Resistant | Yes | Pheno+/Geno- |
| Klebsiella_04 | Tracheal aspirate | AMC | Susceptible | Yes | Pheno-/Geno+ |
| Klebsiella_04 | Tracheal aspirate | AMK | Susceptible | Yes | Pheno-/Geno+ |
| Klebsiella _04 | Tracheal aspirate | ATM | Resistant | Yes | Concordant (+/+) |
| Klebsiella _04 | Tracheal aspirate | CAZ | Susceptible | Yes | Pheno-/Geno+ |
| Klebsiella _04 | Tracheal aspirate | CEZ | Resistant | Yes | Concordant (+/+) |
| Klebsiella _04 | Tracheal aspirate | CIP | Resistant | Yes | Concordant (+/+) |
| Klebsiella _04 | Tracheal aspirate | CRO | Resistant | Yes | Concordant (+/+) |
| Klebsiella _04 | Tracheal aspirate | CTX | Resistant | Yes | Concordant (+/+) |
| Klebsiella _04 | Tracheal aspirate | CXM | Resistant | Yes | Concordant (+/+) |
| Klebsiella _04 | Tracheal aspirate | CXMA | Resistant | Yes | Concordant (+/+) |
| Klebsiella _04 | Tracheal aspirate | FEP | Susceptible | Yes | Pheno-/Geno+ |
| Klebsiella _04 | Tracheal aspirate | GEN | Susceptible | Yes | Pheno-/Geno+ |
| Klebsiella _04 | Tracheal aspirate | MEM | Susceptible | Yes | Pheno-/Geno+ |
| Klebsiella _04 | Tracheal aspirate | SAM | Resistant | Yes | Concordant (+/+) |
| Klebsiella _04 | Tracheal aspirate | SXT | Resistant | Yes | Concordant (+/+) |
| Klebsiella _04 | Tracheal aspirate | TZP | Susceptible | Yes | Pheno-/Geno+ |
| Klebsiella _04 | Tracheal aspirate | FT | Susceptible | Yes | Pheno-/Geno+ |
| Klebsiella _08 | Urine | AMP | Resistant | Yes | Concordant (+/+) |
| Klebsiella _08 | Urine | AMC | Susceptible | Yes | Pheno-/Geno+ |
| Klebsiella _08 | Urine | AMK | Susceptible | Yes | Pheno-/Geno+ |
| Klebsiella _08 | Urine | ATM | Resistant | Yes | Concordant (+/+) |
| Klebsiella _08 | Urine | CAZ | Susceptible | Yes | Pheno-/Geno+ |
| Klebsiella _08 | Urine | CEZ | Resistant | Yes | Concordant (+/+) |
| Klebsiella _08 | Urine | CIP | Resistant | Yes | Concordant (+/+) |
| Klebsiella _08 | Urine | CRO | Resistant | Yes | Concordant (+/+) |
| Klebsiella _08 | Urine | CTX | Resistant | Yes | Concordant (+/+) |
| Klebsiella _08 | Urine | CXM | Resistant | Yes | Concordant (+/+) |
| Klebsiella _08 | Urine | CXMA | Resistant | Yes | Concordant (+/+) |
| Klebsiella _08 | Urine | FEP | Susceptible | Yes | Pheno-/Geno+ |
| Klebsiella _08 | Urine | GEN | Susceptible | Yes | Pheno-/Geno+ |
| Klebsiella _08 | Urine | MEM | Susceptible | Yes | Pheno-/Geno+ |
| Klebsiella _08 | Urine | SAM | Resistant | Yes | Concordant (+/+) |
| Klebsiella _08 | Urine | SXT | Resistant | Yes | Concordant (+/+) |
| Klebsiella _08 | Urine | TZP | Susceptible | Yes | Pheno-/Geno+ |
| Klebsiella _08 | Urine | FT | Susceptible | No | Concordant (-/-) |
| Klebsiella _15 | Blood | AMP | Resistant | Yes | Concordant (+/+) |
| Klebsiella _15 | Blood | AMC | Susceptible | Yes | Pheno-/Geno+ |
| Klebsiella _15 | Blood | AMK | Susceptible | No | Concordant (-/-) |
| Klebsiella _15 | Blood | ATM | Resistant | Yes | Concordant (+/+) |
| Klebsiella _15 | Blood | CAZ | Resistant | Yes | Concordant (+/+) |
| Klebsiella _15 | Blood | CEZ | Resistant | Yes | Concordant (+/+) |
| Klebsiella _15 | Blood | CIP | Susceptible | Yes | Pheno-/Geno+ |
| Klebsiella _15 | Blood | CRO | Resistant | Yes | Concordant (+/+) |
| Klebsiella _15 | Blood | CTX | Resistant | Yes | Concordant (+/+) |
| Klebsiella _15 | Blood | CXM | Resistant | Yes | Concordant (+/+) |
| Klebsiella _15 | Blood | CXMA | Resistant | Yes | Concordant (+/+) |
| Klebsiella _15 | Blood | FEP | Susceptible | Yes | Pheno-/Geno+ |
| Klebsiella _15 | Blood | GEN | Resistant | No | Pheno+/Geno- |
| Klebsiella _15 | Blood | MEM | Susceptible | Yes | Pheno-/Geno+ |
| Klebsiella _15 | Blood | SAM | Resistant | Yes | Concordant (+/+) |
| Klebsiella _15 | Blood | SXT | Resistant | No | Pheno+/Geno- |
| Klebsiella _15 | Blood | TZP | Susceptible | Yes | Pheno-/Geno+ |
| Klebsiella _15 | Blood | FT | Susceptible | No | Concordant (-/-) |
| Klebsiella _17 | Urine | AMP | Resistant | Yes | Concordant (+/+) |
| Klebsiella _17 | Urine | AMC | Resistant | No | Pheno+/Geno- |
| Klebsiella _17 | Urine | AMK | Susceptible | No | Concordant (-/-) |
| Klebsiella _17 | Urine | ATM | Resistant | No | Pheno+/Geno- |
| Klebsiella _17 | Urine | CAZ | Resistant | No | Pheno+/Geno- |
| Klebsiella _17 | Urine | CEZ | Resistant | No | Pheno+/Geno- |
| Klebsiella _17 | Urine | CIP | Resistant | Yes | Concordant (+/+) |
| Klebsiella _17 | Urine | CRO | Resistant | No | Pheno+/Geno- |
| Klebsiella _17 | Urine | CTX | Resistant | No | Pheno+/Geno- |
| Klebsiella _17 | Urine | CXM | Resistant | No | Pheno+/Geno- |
| Klebsiella _17 | Urine | CXMA | Resistant | No | Pheno+/Geno- |
| Klebsiella _17 | Urine | FEP | Resistant | No | Pheno+/Geno- |
| Klebsiella _17 | Urine | GEN | Resistant | No | Pheno+/Geno- |
| Klebsiella _17 | Urine | MEM | Susceptible | Yes | Pheno-/Geno+ |
| Klebsiella _17 | Urine | SAM | Resistant | No | Pheno+/Geno- |
| Klebsiella _17 | Urine | SXT | Resistant | No | Pheno+/Geno- |
| Klebsiella _17 | Urine | TZP | Resistant | No | Pheno+/Geno- |
| Klebsiella _17 | Urine | FT | Resistant | Yes | Pheno+/Geno- |
| Klebsiella _18 | Tracheal aspirate | AMP | Resistant | Yes | Concordant (+/+) |
| Klebsiella _18 | Tracheal aspirate | AMC | Susceptible | Yes | Pheno-/Geno+ |
| Klebsiella _18 | Tracheal aspirate | AMK | Susceptible | No | Concordant (-/-) |
| Klebsiella _18 | Tracheal aspirate | ATM | Resistant | Yes | Concordant (+/+) |
| Klebsiella _18 | Tracheal aspirate | CAZ | Resistant | Yes | Concordant (+/+) |
| Klebsiella _18 | Tracheal aspirate | CEZ | Resistant | Yes | Concordant (+/+) |
| Klebsiella _18 | Tracheal aspirate | CIP | Susceptible | Yes | Pheno-/Geno+ |
| Klebsiella _18 | Tracheal aspirate | CRO | Resistant | Yes | Concordant (+/+) |
| Klebsiella_18 | Tracheal aspirate | CTX | Resistant | Yes | Concordant (+/+) |
| Klebsiella _18 | Tracheal aspirate | CXM | Resistant | Yes | Concordant (+/+) |
| Klebsiella _18 | Tracheal aspirate | CXMA | Resistant | Yes | Concordant (+/+) |
| Klebsiella _18 | Tracheal aspirate | FEP | Resistant | Yes | Concordant (+/+) |
| Klebsiella _18 | Tracheal aspirate | GEN | Susceptible | No | Concordant (-/-) |
| Klebsiella _18 | Tracheal aspirate | MEM | Susceptible | Yes | Pheno-/Geno+ |
| Klebsiella _18 | Tracheal aspirate | SAM | Resistant | Yes | Concordant (+/+) |
| Klebsiella _18 | Tracheal aspirate | SXT | Resistant | No | Pheno+/Geno- |
| Klebsiella _18 | Tracheal aspirate | TZP | Susceptible | Yes | Pheno-/Geno+ |
| Klebsiella _18 | Tracheal aspirate | FT | Susceptible | No | Concordant (-/-) |
| Klebsiella _21 | Tracheal aspirate | AMP | Resistant | No | Pheno+/Geno- |
| Klebsiella _21 | Tracheal aspirate | AMC | Susceptible | No | Concordant (-/-) |
| Klebsiella _21 | Tracheal aspirate | AMK | Susceptible | No | Concordant (-/-) |
| Klebsiella _21 | Tracheal aspirate | ATM | Resistant | No | Pheno+/Geno- |
| Klebsiella _21 | Tracheal aspirate | CAZ | Resistant | No | Pheno+/Geno- |
| Klebsiella _21 | Tracheal aspirate | CEZ | Resistant | No | Pheno+/Geno- |
| Klebsiella _21 | Tracheal aspirate | CIP | Resistant | Yes | Concordant (+/+) |
| Klebsiella_21 | Tracheal aspirate | CRO | Resistant | No | Pheno+/Geno- |
| Klebsiella _21 | Tracheal aspirate | CTX | Resistant | No | Pheno+/Geno- |
| Klebsiella _21 | Tracheal aspirate | CXM | Resistant | No | Pheno+/Geno- |
| Klebsiella _21 | Tracheal aspirate | CXMA | Resistant | No | Pheno+/Geno- |
| Klebsiella _21 | Tracheal aspirate | FEP | Susceptible | No | Concordant (-/-) |
| Klebsiella _21 | Tracheal aspirate | GEN | Susceptible | No | Concordant (-/-) |
| Klebsiella _21 | Tracheal aspirate | MEM | Susceptible | Yes | Pheno-/Geno+ |
| Klebsiella_21 | Tracheal aspirate | SAM | Resistant | No | Pheno+/Geno- |
| Klebsiella _21 | Tracheal aspirate | SXT | Resistant | No | Pheno+/Geno- |
| Klebsiella _21 | Tracheal aspirate | TZP | Susceptible | No | Concordant (-/-) |
| Klebsiella _21 | Tracheal aspirate | FT | Susceptible | No | Concordant (-/-) |
| Klebsiella _23 | Pus swab | AMP | Resistant | Yes | Concordant (+/+) |
| Klebsiella _23 | Pus swab | AMC | Susceptible | Yes | Pheno-/Geno+ |
| Klebsiella _23 | Pus swab | AMK | Susceptible | Yes | Pheno-/Geno+ |
| Klebsiella _23 | Pus swab | ATM | Resistant | Yes | Concordant (+/+) |
| Klebsiella _23 | Pus swab | CAZ | Resistant | Yes | Concordant (+/+) |
| Klebsiella _23 | Pus swab | CEZ | Resistant | Yes | Concordant (+/+) |
| Klebsiella _23 | Pus swab | CIP | Resistant | Yes | Concordant (+/+) |
| Klebsiella _23 | Pus swab | CRO | Resistant | Yes | Concordant (+/+) |
| Klebsiella _23 | Pus swab | CTX | Resistant | Yes | Concordant (+/+) |
| Klebsiella_23 | Pus swab | CXM | Resistant | Yes | Concordant (+/+) |
| Klebsiella _23 | Pus swab | CXMA | Resistant | Yes | Concordant (+/+) |
| Klebsiella _23 | Pus swab | FEP | Susceptible | Yes | Pheno-/Geno+ |
| Klebsiella _23 | Pus swab | GEN | Susceptible | Yes | Pheno-/Geno+ |
| Klebsiella _23 | Pus swab | MEM | Susceptible | Yes | Pheno-/Geno+ |
| Klebsiella _23 | Pus swab | SAM | Resistant | Yes | Concordant (+/+) |
| Klebsiella _23 | Pus swab | SXT | Resistant | Yes | Concordant (+/+) |
| Klebsiella _23 | Pus swab | TZP | Susceptible | Yes | Pheno-/Geno+ |
| Klebsiella _23 | Pus swab | FT | Susceptible | No | Concordant (-/-) |
