# Supplementary material for: Phenotypic and genomic profiling of multidrug-resistant Escherichia coli and Klebsiella pneumoniae isolated from Intensive Care Unit patients in Kenya
Source: BMC Microbiol. 2026 Mar 23;26:419. doi: 10.1186/s12866-026-04880-5 (PMC13130711; doi:10.1186/s12866-026-04880-5)
Supplement: Supplementary file 3 — Supplementary Material 3. [file 12866_2026_4880_MOESM3_ESM.docx]

#### **Supplementary Table S3:** Quality assessment tool (QUAST) assembly statistics.

| Isolate ID | Contigs | Largest contig | Total length | GC% | N50 | L50 |
| --- | --- | --- | --- | --- | --- | --- |
| E. coli_KNH_02 | 18 | 1201859 | 5145239 | 50.91 | 822361 | 3 |
| E. coli_KNH_03 | 13 | 3481314 | 5163745 | 50.87 | 3481314 | 1 |
| E. coli_KNH_05 | 3 | 5128195 | 5305016 | 50.53 | 5128195 | 1 |
| E. coli_KNH_06 | 9 | 5022444 | 5567494 | 50.68 | 5022444 | 1 |
| E. coli_KNH_07 | 11 | 2232766 | 5598465 | 50.68 | 978482 | 2 |
| E. coli_KNH_09 | 38 | 5105422 | 5578956 | 51.00 | 5105422 | 1 |
| E. coli_KNH_10 | 14 | 1321730 | 5108385 | 50.89 | 699663 | 3 |
| E. coli_KNH_11 | 11 | 1257921 | 5232304 | 50.56 | 923511 | 3 |
| E. coli_KNH_12 | 26 | 751536 | 4781818 | 50.63 | 237831 | 6 |
| E. coli_KNH_13 | 26 | 751536 | 4781818 | 50.63 | 237831 | 6 |
| E. coli_KNH_14 | 26 | 751536 | 4781818 | 50.63 | 237831 | 6 |
| E. coli_KNH_16 | 70 | 362814 | 6797296 | 50.48 | 127062 | 15 |
| E. coli_KNH_19 | 10 | 2360078 | 5230247 | 50.61 | 1534297 | 2 |
| E. coli_KNH_20 | 27 | 5111592 | 6544414 | 52.17 | 5111592 | 1 |
| E. coli_KNH_22 | 4 | 4244064 | 4770413 | 50.65 | 4244064 | 1 |
| Klebsiella_KNH_04 | 4 | 4193458 | 5614787 | 57.02 | 4193458 | 1 |
| Klebsiella_KNH_08 | 22 | 1476282 | 5607363 | 57.01 | 556518 | 4 |
| Klebsiella_KNH_15 | 23 | 1714740 | 5597548 | 56.99 | 723753 | 3 |
| Klebsiella_KNH_17 | 36 | 382217 | 3327415 | 57.43 | 108853 | 10 |
| Klebsiella_KNH_18 | 31 | 1332335 | 5606300 | 56.44 | 379192 | 4 |
| Klebsiella_KNH_21 | 30 | 566395 | 4280941 | 57.51 | 272311 | 6 |
| Klebsiella_KNH_23 | 24 | 738245 | 4716196 | 57.04 | 279261 | 6 |

Contigs: long stretches of DNA sequence assembled from shorter DNA fragments; GC content: percentage of nucleotides in the genome that are either guanine (G) or cytosine (C); N50 length: length of the shortest contig such that 50% of the genome is contained in contigs of that length or longer; L50 is the number of contigs required to cover 50% of the genome.
