# Supplementary material for: Phenotypic and genomic profiling of multidrug-resistant Escherichia coli and Klebsiella pneumoniae isolated from Intensive Care Unit patients in Kenya
Source: BMC Microbiol. 2026 Mar 23;26:419. doi: 10.1186/s12866-026-04880-5 (PMC13130711; doi:10.1186/s12866-026-04880-5)
Supplement: Supplementary file 4 — Supplementary Material 4. [file 12866_2026_4880_MOESM4_ESM.docx]

**Supplementary Table 4A: Distribution of plasmid replicons and antimicrobial resistance genes among *E. coli* isolates.**

| **ID** | **ST** | **Sample** | **Plasmids (n)** | **Key resistance genes** | **AMR genes (n)** |
| --- | --- | --- | --- | --- | --- |
| E02 | 10 | U | IncF-family, IncI1-1(α) (4) | *bla_CTX-M-15_, bla_OXA-1_, aac(6')-Ib, gyrA, tetB, sul1/2* | 13 |
| E03 | 44 | T | IncF-family, IncI1-1(α), col156 (5) | *bla_CTX-M-15_, bla_OXA-1_, gyrA, dfrA17, erm(B)* | 13 |
| E05 | 648 | P | IncF-family (3) | *bla_CTX-M-15_, bla_OXA-1_, blaTEM-1, dfrA17, erm(B)* | 14 |
| E06 | 131 | T | IncF-family, IncI1-1(α), col156, IncY (6) | *bla_CTX-M-15_, bla_OXA-534_, aph(3')-Ia, tetA/B, dfrA17* | 16 |
| E07 | 131 | T | IncF-family, IncI1-1(α) (4) | *bla_CTX-M-15_, bla_TEM-1_, aph(3')-Ia, sul1/2, erm(B)* | 16 |
| E09 | 131 | U | IncF-family, IncI1-1(α) (4) | *bla_CTX-M-15_, bla_OXA-1_, dfrA17, erm(B)* | 13 |
| E10 | 10 | U | IncF-family, IncI1-1(α), col156 (5) | *bla_CTX-M-15_, bla_OXA-1_, aph(3")-Ib, sul1/2, dfrA17* | 14 |
| E11 | 648 | U | IncF-family, col156 (4) | *bla_CTX-M-15_, bla_TEM-1_, oqxA, aph(6)-Id, dfrA17* | 16 |
| E12 | 648 | B | IncF-family (3) | *bla_CTX-M-15_, bla_OXA-1_, gyrA* | 8 |
| E13 | 648 | U | IncF-family (3) | *bla_CTX-M-15_, bla_OXA-1_, dfrA17, erm(B)* | 13 |
| E14 | 648 | T | IncF-family, IncI1-1(α) (3) | *bla_CTX-M-15_, bla_TEM-1_, bla_OXA-1_* | 9 |
| E16 | ND | T | IncF-family (1) | *bla_CTX-M-15_, bla_OXA-534_, dfrA17, erm(B)* | 12 |
| E19 | 14 | T | IncF-family, IncI1-1(α) (3) | *bla_CTX-M-15_, aph(3')-Ia, tetA/B, dfrA17, erm(B)* | 15 |
| E20 | 23 | T | IncF-family, IncI1-1(α), col156 (4) | *bla_CTX-M-15_, bla_CMY-2_, bla_OXA-534_, aac(3)-IId, dfrA17* | 15 |
| E22 | ND | T | IncF-family, col(BS512) (2) | *bla_CTX-M-15_, bla_TEM-30_, oqxB, fosA, arr-3, dfrA14* | 12 |

Abbreviations: ST, sequence type; ND, not determined; B, blood; P, pus swab; T, tracheal aspirate; U, urine. IncF-family-family includes IncF-IA, IncF-IB, and/or IncF-II replicons. Key resistance genes represent clinically significant determinants; complete gene lists are available in Supplementary Table S1.

Sample IDs

| E. coli_KNH_02 | E02 |
| --- | --- |
| E. coli_KNH_03 | E03 |
| E. coli_KNH_05 | E05 |
| E. coli_KNH_06 | E06 |
| E. coli_KNH_07 | E07 |
| E. coli_KNH_09 | E09 |
| E. coli_KNH_10 | E10 |
| E. coli_KNH_11 | E11 |
| E. coli_KNH_12 | E12 |
| E. coli_KNH_13 | E13 |
| E. coli_KNH_14 | E14 |
| E. coli_KNH_16 | E16 |
| E. coli_KNH_19 | E19 |
| E. coli_KNH_20 | E20 |
| E. coli_KNH_22 | E22 |

**Supplementary Table 4B: Distribution of plasmid replicons and antimicrobial resistance genes among *K. pneumoniae* isolates.**

| **ID** | **ST** | **Sample** | **Plasmids (n)** | **Key resistance genes** | **AMR genes (n)** |
| --- | --- | --- | --- | --- | --- |
| K04 | ND | B | IncR (1) | *bla_CTX-M-15_, bla_TEM-1_, bla_SHV-1_, aph(6)-Id, sul2, OqxA, mph(A), dfrA12, fosA, qnrB1* | 14 |
| K08 | ND | P | IncFIB(K) (1) | *bla_CTX-M-15_, bla_TEM-1_, bla_SHV-1_, aph(3')-Ib, catA1, sul2, tetA, aadA2, OqxA, mph(A), dfrA14* | 14 |
| K15 | 405 | T | IncFIB(K) (1) | *bla_CTX-M-15_, bla_TEM-1_, bla_SHV-1_, aac(3)-IId, aph(3')-Ib, sul2, tetB, OqxA, mph(A), dfrA14, fosA, qnrB1* | 15 |
| K17 | ND | T | IncI1-1(α) (1) | *bla_CTX-M-15_, bla_TEM-1_, bla_SHV-1_, aph(3')-Ib, aac(3)-IId, aph(3')-Ia, sul1, sul2, tetA, tetB, aadA2, OqxA, mph(A), dfrA12, dfrA14, fosA* | 18 |
| K18 | ND | T | None (0) | *bla_CTX-M-15_, bla_TEM-1_, bla_SHV-1_, sul2, OqxA, mph(A), dfrA12, fosA, qnrB1* | 11 |
| K21 | ND | U | IncFIB(K), IncFIA(PBK30683), IncHI1B(pNDM-MAR) (3) | *bla_CTX-M-15_, bla_TEM-1_, bla_SHV-1_, aph(6)-Id, sul2, OqxA, mph(A), aph(6)-Id, dfrA12, fosA, qnrB1* | 13 |
| K23 | ND | U | None (0) | *bla_CTX-M-15_, bla_SHV-1_, catA1, sul2, tetB, OqxA, mph(A), dfrA12, fosA* | 11 |

Abbreviations: ST, sequence type; ND, not determined (untypeable); Sample types: B, blood; P, pus swab; T, tracheal aspirate; U, urine. Plasmid replicon count shown in parentheses. Key resistance genes represent clinically significant determinants; complete gene lists are available in Supplementary Table S1.

Sample IDs

| Klebsiella_KNH_04 | K04 |
| --- | --- |
| Klebsiella_KNH_08 | K08 |
| Klebsiella_KNH_15 | K15 |
| Klebsiella_KNH_17 | K17 |
| Klebsiella_KNH_18 | K18 |
| Klebsiella_KNH_21 | K21 |
| Klebsiella_KNH_23 | K23 |
